# Active querying approach to epidemic source detection on contact networks

**DOI:** 10.1038/s41598-023-38282-8

**Published:** 2023-07-13

**Authors:** Martin Sterchi, Lorenz Hilfiker, Rolf Grütter, Abraham Bernstein

**Affiliations:** 1 Department of Informatics, University of Zurich, 8050 Zurich, Switzerland; 2 School of Business, University of Applied Sciences and Arts FHNW, 4600 Olten, Switzerland; 3 Swiss Federal Research Institute WSL, 8903 Birmensdorf, Switzerland; 4 Institute of Mathematical Statistics and Actuarial Science, University of Bern, 3012 Bern, Switzerland

**Keywords:** Computer science, Epidemiology, Complex networks

## Abstract

The problem of identifying the source of an epidemic (also called *patient zero*) given a network of contacts and a set of infected individuals has attracted interest from a broad range of research communities. The successful and timely identification of the source can prevent a lot of harm as the number of possible infection routes can be narrowed down and potentially infected individuals can be isolated. Previous research on this topic often assumes that it is possible to observe the state of a substantial fraction of individuals in the network before attempting to identify the source. We, on the contrary, assume that observing the state of individuals in the network is costly or difficult and, hence, only the state of one or few individuals is initially observed. Moreover, we presume that not only the source is unknown, but also the duration for which the epidemic has evolved. From this more general problem setting a need to query the state of other (so far unobserved) individuals arises. In analogy with active learning, this leads us to formulate the *active querying* problem. In the active querying problem, we alternate between a source inference step and a querying step. For the source inference step, we rely on existing work but take a Bayesian perspective by putting a prior on the duration of the epidemic. In the querying step, we aim to query the states of individuals that provide the most information about the source of the epidemic, and to this end, we propose strategies inspired by the active learning literature. Our results are strongly in favor of a querying strategy that selects individuals for whom the disagreement between individual predictions, made by all possible sources separately, and a consensus prediction is maximal. Our approach is flexible and, in particular, can be applied to static as well as temporal networks. To demonstrate our approach’s practical importance, we experiment with three empirical (temporal) contact networks: a network of pig movements, a network of sexual contacts, and a network of face-to-face contacts between residents of a village in Malawi. The results show that active querying strategies can lead to substantially improved source inference results as compared to baseline heuristics. In fact, querying only a small fraction of nodes in a network is often enough to achieve a source inference performance comparable to a situation where the infection states of all nodes are known.

## Introduction

The spread of infectious diseases is one of the main global threats to humans, animals, and the economy. The pandemic caused by the SARS-CoV-2 virus has painfully demonstrated what happens if a local outbreak of an infectious disease cannot be contained. However, the spread of infectious diseases is not limited to humans. It is, for example, also a common occurrence in networks of livestock holdings, where a pathogen is spread via livestock (e.g., pig) movements between holdings^1–4^. From a scientific perspective, disease spread in livestock is of particular interest, since data on the underlying contact networks are often more readily available than for humans. Moreover, from a *One Health*^5^ perspective the monitoring of livestock movements makes sense, considering that animal and human health are closely interrelated.

The increased availability of contact network data, in general, has led to a variety of studies of spreading processes on networks^6^. One topic that has received a lot of attention and has prompted at least one survey article^7^ is the identification of the source node of an epidemic (also called *patient zero*), or more generally of a propagation process. Being able to identify or detect the source of a local outbreak can play an important role in contact tracing efforts^8^. Once we know the source of an outbreak, it becomes easier to find all possible downstream contacts and take appropriate mitigation measures.

### Source inference

The source inference problem has been formally introduced in seminal work by Shah and Zaman^9^ in the context of computer virus spreading on *static* networks. The authors show that for regular trees the likelihood of a node being the source is proportional to the number of infection sequences, originating from that source, that are consistent with a given infection tree. This number of infection sequences can be seen as a node centrality measure and the authors propose calling it *rumor centrality*. Another centrality-based approach that is worth mentioning is the *Jordan centrality*, where the most likely source is determined as the node that minimizes the maximum distance to other infectious nodes^10^. A distance-based approach has also been suggested in the context of metapopulation models^11^, where the authors estimate the most likely source as the node which renders the set of observed infected nodes most “concentric” with respect to an effective distance. Yet another purely topological approach estimates the source as the node which produces the (temporal) Steiner tree of lowest cost, where the cost is measured as the sum over the tree’s edge weights^12^. Unlike many topological approaches, this method works both on static and temporal networks. Moreover, the last two methods are both agnostic with respect to the spreading dynamics.

A different, more probabilistic branch of literature^13,14^ uses well-known epidemic propagation models to compute individual node state probabilities, i.e., the probabilities that some node is in a given state (e.g., infectious) at some time, given a source node and a start time of the outbreak. The most likely source is then inferred by maximum likelihood (or Bayesian) estimation. Typically, a strong node independence assumption is employed both to make the computation of the node state probabilities tractable and to allow the computation of the source likelihood as a product of individual node state probabilities. In the context of computing the node state probabilities, this simplification is known as *individual-based approximation* (IBA) and has been widely applied both on static^15–17^ and temporal networks^14^. Alternatively, the resulting approximation error can be avoided, at the cost of a higher computational expense, by computing the individual node state probabilities via Monte-Carlo simulations. This is the route that will be taken in the present paper, with the node independence assumption only being used to approximate the source likelihood as a product of the individual node state probabilities.

One may wonder why the node independence assumption is necessary at all, seeing as it may be possible to directly simulate outbreaks and count the number of times the simulated outcome is equal to the observed outcome. However, a naive implementation of such an approach becomes infeasible as simulated outcomes are increasingly unlikely to match the observed outcome when the set of observed nodes grows. As a workaround, Antulov-Fantulin et al.^18^ propose to measure the similarity (as opposed to equality) between the observed outcome and simulated outcomes from a given source. The higher the similarity for a given source, the more a simulation outcome contributes to the likelihood of that source. An entirely different approach has been developed by Braunstein and Ingrosso^19^, who use a form of *belief propagation* for a continuous-time epidemic model on temporal networks in order to model the likelihood. Although their approach does not rely on the node independence assumption, it still constitutes only an approximation for all networks except for trees.

### Active querying

All the studies mentioned in the previous paragraphs assume that the states (susceptible, infectious, recovered, etc.) of all individuals or a substantial fraction of individuals in the contact network are known, which, as the COVID pandemic has shown, is often not realistic. A number of studies^20–26^ have looked at the problem of optimal selection of a limited number of observers for spreading processes on static networks. However, these studies assume the observers to be selected a priori before any observation has been made. Moreover, they rely on knowing the exact infection times, and in some cases even the infecting neighbors of the observers.

An ultimately more realistic assumption is the scenario in which, at first, only the state of one node is observed. This changes the problem drastically: should we just use the initially observed node to determine the most likely source? Or should we gather more information about the outbreak by querying the state of other nodes such that we can narrow down the set of likely sources? In this paper, we focus on the second question and we will define the *active querying* problem in the context of the well-known *susceptible-infectious-recovered* (SIR) spreading model. The active querying problem is concerned with the decision of which nodes to query about their state in order to learn as much as possible about the true source of the epidemic. Zejnilović et al.^27^ consider a related problem of sequential observer selection in the context of a deterministic spreading process on static networks. Spinelli et al.^25^ also study sequential observer selection on static networks, but knowledge of infection times is crucial to their approach. A major drawback of both methods is that they do not apply to temporal networks.

In this paper, we propose an iterative two-step approach. First, we perform an inference step based on the state of the initially observed node, which yields a bivariate posterior distribution over possible source nodes and possible durations between the start of the epidemic and the time of the first detection. Second, we pick an unobserved node according to some selection strategy and query its state (susceptible, infectious, or recovered). The newly observed node will augment the evidence about the outbreak and may improve the inference about the true source node (and the true duration of the epidemic). We then iterate this inference-querying cycle until some maximal number of queries is reached. For the querying step, we propose different active querying strategies that are based on ideas borrowed from the active learning literature^28,29^. These strategies are based entirely on the node state probabilities and therefore are flexible with respect to the type of underlying network. Importantly, they can be applied to directed networks and temporal networks. The active querying strategies will be compared to simple baseline strategies such as random querying.

### Contributions

In summary, the contributions of this paper are as follows:

- We revisit the well-known source inference problem where all node states are observed at some specified time and analyze the source detection performance at different stages of the epidemic outbreak. In our problem setting both the source and the starting time of the epidemic are unknown. The results of this analysis serve as a benchmark for the querying results.
- We introduce the active querying problem in the context of source inference and propose a set of active querying strategies which are inspired by the work on active learning.
- We evaluate our active querying approach on three real-world (timestamped) contact networks: a network of pig movements in Switzerland, a network of assumed sexual contacts between sex workers and their clients that was derived from an internet community^30,31^, and a network of contacts between 86 residents of a village in Malawi^32^. We demonstrate how our computational approach for the active querying problem can lead to swift and efficient containment of an epidemic if the outbreak is queried in a smart (active) way. In addition, the Supplementary Information provides results on well-known static network models.

Before we present and discuss the results of our work, we will provide some preliminary notions and formally introduce the inference-querying cycle we propose.

## Preliminaries

In this section, we will formally present the notion of networks as well as the SIR spreading model that forms the basis of our work. We will then formulate the problem that our work aims to solve.

### Contact networks

The spreading of an infectious disease occurs through (physical) contact between people or animals. Such contacts can be conceptualized as a network with the individuals being nodes and the contacts being edges. We denote the network (or graph) as *G*(*V*, *E*), where *V* corresponds to the set of nodes (or vertices) of size $N=|V|$ and *E* corresponds to the set of edges of size |*E*|. The study of propagation processes on networks has traditionally focused on static networks, where contacts between nodes persist over time^33^. In this case, an (undirected) edge is simply defined as a tuple $(v, u) \in E$, where $v, u \in V$ and $(v, u) = (u, v)$.

Recently, the study of temporal networks has received more attention^12,14,18,19^, in part because such data have increasingly become available. In our work, a (temporal) edge is defined as a 3-tuple $(v, u, t) \in E$, where $t \in \mathbb N$ corresponds to a discrete timestamp defining the time when the nodes *v* and *u* were in contact. The propagation of the disease between nodes is constrained by the temporal sequence of contacts. For example, the existence of a spreading cascade between nodes *A* and *C* via *B* does not only depend on the existence of edges $(A, B, t_1)$ and $(B, C, t_2)$ but also on whether $t_1 < t_2$. Note the strict inequality in the last expression as we assume that a node cannot get infected and further infect other nodes within a single time step.

### SIR spreading model

The *susceptible-infectious-recovered* (SIR) model on a (static) network^17^ assumes that at each point in time *t*, a node is in one of three possible states (often called compartments): susceptible (S), infectious (I), or recovered (R). The dynamic process that is described by SIR is irreversible with two possible state transitions: susceptible nodes becoming infectious ($S \rightarrow I$) at rate $k\beta$ if *k* neighbors in the network are in the infectious state and infectious nodes becoming recovered ($I \rightarrow R$) at rate $\mu$ (independently of the states of other nodes). For the analysis of the static networks (see Supplementary Information) we model the transmission and recovery events as Poisson processes with the rates $\beta$ and $\mu$ being constant, thereby implicitly assuming that time is continuous. For the analysis of the empirical temporal contact networks, we will use a discrete-time version of the SIR model. Instead of rates, we will use state transition probabilities that describe the likelihood of transitioning to another state during one time step. Note that the aforementioned restriction that spreading cascades cannot occur within a single time step makes the SIR model effectively a *susceptible-exposed-infectious-recovered* (SEIR) model with nodes being in the exposed state for a period shorter than the network’s resolution.^34^

### Problem formulation

Suppose we are given a contact network *G*(*V*, *E*), where contacts are either static or temporal. Furthermore, we assume that a disease spreads according to a SIR model with known parameters $\beta$ and $\mu$. The spreading process initiates from *one* source node denoted as $q_0$ at some time $t_0$ and continues for a period of time *T* until the process is detected at time $t_0 + T$. For the analysis of the static networks (see Supplementary Information), we assume that only the true source $q_0$ is unknown to us and that $t_0$ is known. For the analysis of the empirical temporal contact networks, we relax this assumption and assume that both $q_0$ and $t_0$ (and consequently *T*) are unknown. We model the source of the epidemic and the duration of the epidemic until the first observation as discrete random variables *Q* and *T*, respectively. The state space of *Q* consists of the set of all nodes *V*, and the state space of *T* is the set $\mathscr {T} = \{0, \dots , K\}$ for some sufficiently large integer *K*.

At each point in time, every node $v \in V$ is in one of the three possible states of the SIR model. We denote the state of node *v* at any time $t > t_0$ as a random variable $X_v(t)$ that can take three possible values, $X_v(t) \in \{S, I, R\}$. At some time $t_1 = t_0 + T$, we make an observation about the states of the nodes in the network. We will distinguish two cases of observations: (1) we observe the state of all nodes in the network, which corresponds to a problem that has been studied extensively in related work^14,18,19^ and (2) we initially observe the state of only one infectious or recovered node and then observe the state of additional nodes one at a time. In both cases, we denote the set of observed nodes as $O_{t_1} \subseteq V$ and the corresponding evidence as $E_{t_1} = \{(v, x_v(t_1)): v \in O_{t_1}\}$. In the first case, we simply aim to infer the maximum a posteriori (MAP) node, after marginalizing the posterior over *T*. By contrast, in the second case, we alternate an inference and query step. Queried nodes and their states are incrementally added to $E_{t_1}$ until we eventually have enough nodes to confidently determine which node may be the true source node $q_0$. Hence, the main problem that we attempt to solve is how to decide which unobserved nodes to query in order to find the true source node $q_0$ (and the duration of the epidemic) in an efficient way, i.e., with as few queries as possible. We assume for simplicity that all queries are made at time $t_1$, but performed sequentially.

## Inference and querying

In this section, we describe the three main components of our approach. First, we introduce the inference mechanism. Second, we describe the method we use to compute individual node state probabilities given a source node and a starting time. Finally, we present three active querying strategies.

### Inference

We assume that only the SIR parameters of the propagation process are known. Hence, our problem consists of inferring both the source node *Q* and the duration until the first observation *T*, given the evidence $E_{t_1}$. Note that once we infer *T*, we can also infer the starting time with $t_1 - T$. As a consequence, we attempt to calculate a joint posterior distribution $P(Q, T \, | \, E_{t_1})$ over all nodes in *V* and all durations $T \in \mathscr {T}$. We suppress the dependence on the fixed SIR parameters $\beta$ and $\mu$ in order to keep the notation uncluttered. Assuming a-priori independence between *Q* and *T* and a uniform prior over *Q*, the posterior distribution can be written as follows:

1 $$\begin{aligned} P(Q, T \, | \, E_{t_1}) = \frac{P(E_{t_1} \, | \, Q, T) \cdot P(T)}{\sum _{q \in V} \sum _{d \in \mathscr {T}} P(E_{t_1} \, | \, Q' = q, T' = d) \cdot P(T' = d)} \end{aligned}$$

The problem with the inference expression above is that computing the likelihood $P(E_{t_1} \, | \, Q, T)$, i.e., the probability of observing $E_{t_1}$ given a possible source node *Q* and duration until first observation *T*, is not straightforward because the states of nodes are not independent of each other in a networked system. For a continuous-time SIR process on static networks of size *N*, the exact likelihood could be computed by modeling the problem as a continuous-time Markov chain (see Supplementary Information for an example). However, this would require $3^N-1$ master equations, which is not practical except for very small networks^17^. Therefore, the likelihood is usually computed using simplifying assumptions or by resorting to Monte-Carlo simulations. One common simplification is to assume that node states are independent of each other, which, in the context of networks, is called the *mean-field-like approximation*^13^. It allows us to compute the likelihood simply as the product of individual node state probabilities. Although a strong assumption, it has been shown that inference based on this independence assumption generally leads to good estimates for the source of an epidemic process on static^13^ and on temporal networks^14^.

Given this independence assumption, the likelihood $P(E_{t_1} \, | \, Q=q, T=d)$ can be written as the product over individual node state probabilities $P_{q,d}(X_v(t)) {:}{=}P(X_v(t) \, | \, Q=q, T=d)$. This allows for efficient computation of the likelihood, provided the individual node state probabilities have been computed beforehand. In order to avoid numerical underflow, we apply the log-sum-exp trick to compute the posterior distribution in Eq. (1) (see Supplementary Information).

Once we have computed the posterior in Eq. (1), we can first marginalize out *T* and then determine the most likely source as the node *q* with the maximal marginal posterior probability, i.e.,

2 $$\hat{q} = \mathop {{\text{argmax}}}\limits_{{q \in V}} \left[ {\sum\limits_{{d \in {\mathscr{T}}}} P (Q,T = d|{\mkern 1mu} E_{{t_{1} }} )} \right].$$

Note that the proposed inference mechanism is conceptually equivalent to the Naive Bayes classifier with priors on *Q* and *T*, where the feature vector consists of $|O_{t_1}|$ categorical components and the target classes are the elements in *V*.

### Node state probabilities

This subsection is concerned with estimating the individual node state probabilities $P_{q,d}(X_v(t))$, as they constitute the key component for computing the (approximate) likelihood. In our approach, we use Monte-Carlo simulations to compute the node state probabilities, given a source and a duration until the first observation. More specifically, we stochastically simulate a large number *n* of spreading processes up to time $t_1$, originating from source $Q=q$ and using a duration until the first observation $T=d$. If $n_{v,\,I}(t)$ describes the number of simulations for which node *v* is infectious at time *t* (i.e., $X_v(t)=I$), then we can estimate the probability of node *v* being infectious at time *t*, given source *q* and duration *d*, as $P_{q,d}(X_v(t)=I) = n_{v,\,I}(t)\,/\,n$. Analogously, we can compute the probabilities of node *v* being susceptible or recovered.

Research on epidemic simulation approaches has been an active field in recent years^34,35^, and fast event-driven approaches have been implemented for static networks^36^ and for temporal networks^34^. These new implementations make it possible to run extensive Monte-Carlo simulations on realistic networks in a reasonable amount of time. In the context of source detection, Monte-Carlo methods have only been applied in the work of Antulov-Fantulin et al.^18^, Dutta et al.^37^, and in the simple case of a deterministic SI-model where a mathematical trick yields a shortcut for the simulations^21^. We use Holme’s implementation of an event-driven approach for temporal networks, which has a worst-case time complexity per simulation run of $\mathcal {O}(N \cdot \text{ log } \; N \cdot \text{ log } \; C)$ for sparse networks with *C* being the maximum number of contacts between the same pair of nodes.^34^ However, as Holme^34^ notes, realistic networks, especially temporal ones, are typically fragmented, which, together with realistic epidemic parameters, often leads to rather small outbreaks. As a consequence, run times may be much faster in practice than the worst-case scenario suggests. As our approach requires running *n* simulations for each source-duration pair, we can write the overall time complexity of the simulation approach as $\mathcal {O}(n \cdot N^2 \cdot \text{ log } \; N \cdot \text{ log } \; C)$, ignoring the durations since $|\mathscr {T}|$ is typically smaller than *N*. In the Supplementary Information, we review two other approaches to the task of computing node state probabilities and compare them to the Monte-Carlo approach we use here.

### Active querying

Thus far, we have described the inference step and the computation of the node state probabilities. To conclude this section, we will now introduce five different strategies that help us decide which unobserved nodes to query. Crucially, the evidence $E_{t_1}$ will be extended every time an unobserved node is queried and its state at $t_1$ becomes known. After each query step, we can recompute the posterior distribution and the next querying step will then be based on the updated posterior. We will consider three active and two baseline querying strategies.

#### Uncertainty sampling: UCTY strategy

Intuitively, an active querying strategy should aim to query nodes whose states we are most uncertain about. This naturally leads to measuring the uncertainty about a node’s state with the help of entropy. For this, we define the overall node state probability for a node *v* as

3 $$\begin{aligned} P(X_v(t_1)) {:}{=}\sum _{q \in V} \sum _{d \in \mathscr {T}} P_{q,d}(X_v(t_1)) \cdot P(Q=q, T=d \, | \, E_{t_1}). \end{aligned}$$

This is a form of Bayesian model averaging where we use the posterior distribution to weigh the individual node state probabilities. With the expression in Eq. (3), we can compute each node’s entropy:

4 $$\begin{aligned} H_v(P) {:}{=}~~ -\sum _{x \in \{S,I,R\}} P(X_v(t_1) = x) \cdot \text{ log }\, P(X_v(t_1) = x). \end{aligned}$$

Finally, we query the (unobserved) node with the largest entropy, i.e.,

5 $$v^{*} = \mathop{{\text{argmax}}}\limits_{v \in V{\backslash }O_{{t_{1} }} } \;H_{v} (P).$$

In the active learning community this is known as uncertainty sampling^28^, which we refer to as the *UCTY strategy*.

#### Activated nodes sampling: MAXP strategy

The second active strategy that we propose attempts to query nodes that are infectious or recovered, i.e., *activated* nodes. The rationale is that querying an activated node is more informative than querying a susceptible node, especially in small outbreaks with few activated nodes. For this, we simply query the (unobserved) node with the largest overall probability of being infectious or recovered, i.e.,

6 $$v^{*} = \mathop{{\text{argmax}}}\limits_{v \in V{\backslash }O_{{t_{1} }} }{\mkern 1mu} \left[ {1 - P(X_{v} (t_{1} ) = S)} \right].$$

We refer to this as the *MAXP strategy*.

#### Disagreement sampling: AKLD strategy

The third active querying strategy we propose is, yet again, motivated by ideas from the active learning literature, in particular the *query-by-committee* literature^28,38^. The general idea is to query a node *v* for which the different source-duration pairs (*q*, *d*) have strongly differing “opinions” about *v*’s state. We define a source-duration pair’s opinion about some node *v* as the individual node state probability $P_{q,d}(X_v(t_1))$. Thus, we aim to query controversial nodes. For a given (unobserved) node *v* and each possible source $q \in V$ and duration $d \in \mathscr {T}$, we can compute the Kullback-Leibler (KL) divergence between the node state distribution given *q* and *d* and the overall node state distribution as computed in Eq. (3), i.e.,

7 $$\begin{aligned} \begin{aligned} D_v({P_{q,d}}\Vert {P})&= \sum _{x \in \{S,I,R\}} P_{q,d}(X_v(t_1) = x) \cdot \text{ log } \left( \frac{P_{q,d}(X_v(t_1) = x)}{P(X_v(t_1) = x)}\right) \\ {}&= \sum _{x \in \{S,I,R\}} P_{q,d}(X_v(t_1) = x) \cdot \text{ log } \; P_{q,d}(X_v(t_1) = x) ~~ - \sum _{x \in \{S,I,R\}} P_{q,d}(X_v(t_1) = x) \cdot \text{ log } \; P(X_v(t_1) = x) \\ {}&= -H_v(P_{q,d}) + H_v(P_{q,d}, P). \end{aligned} \end{aligned}$$

Hence, the KL-divergence corresponds to the difference between the cross-entropy $H_v(P_{q,d}, P)$ (for the two distributions $P_{q,d}$ and *P*) and the entropy $H_v(P_{q,d})$ for the individual node state distribution $P_{q,d}$. We then select the unobserved node with the maximum average KL-divergence:

8 $$v^{*} = \mathop {{{\text{argmax}}}}\limits_{v \in V{\backslash }O_{{t_{1} }}} \left[ {\sum\limits_{{q \in V}} {\sum\limits_{{d \in {\mathscr{T}}}} P } (Q = q,T = d|{\mkern 1mu} E_{{t_{1} }} ) \cdot \left[ {H_{v} (P_{{q,d}} ,P) - H_{v} (P_{{q,d}} )} \right]} \right].$$

We refer to this as the *AKLD strategy*.

In Algorithm (1), we provide pseudo-code for the AKLD strategy. It is easy to see that AKLD has a (worst-case) complexity of $\mathcal {O}(N^2)$ (provided that the overall probability distributions $P(X_v(t_1))$ have been computed beforehand). By contrast, UCTY and MAXP simply have a complexity of $\mathcal {O}(N)$.
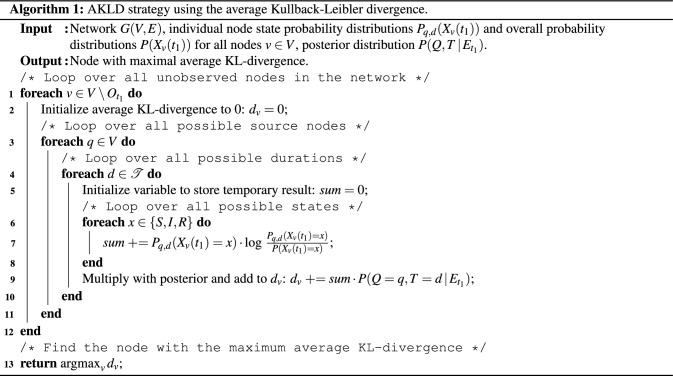


#### Baseline strategies: RANDOM and ONE-HOP

As a benchmark for the active querying strategies, we will consider two simple baseline strategies. The first baseline strategy, *RANDOM*, samples the next node to be queried uniformly at random from the set of all unobserved nodes that can be reached by any of the possible sources at the earliest possible starting time. The second baseline strategy, *ONE-HOP*, selects nodes to be queried uniformly at random among the direct neighbours of observed, activated nodes. Only neighbors with contact times between the earliest possible starting time and the inference time are considered. Note that, for all five strategies, we break ties by picking a node at random.

## Results

This section has been divided into four parts. First, we present the three networks used to evaluate our approach. Second, we illustrate with an example how our approach works for a network of animal transports. Third, we present the results for the source inference problem where the states of all nodes in the network are observed. These results will serve as a benchmark for the querying results, as observing all node states corresponds to the maximum amount of information that can be obtained. Finally, in the fourth part, we present the active querying results as compared to the fully observed inference performance. A good querying strategy is one that approaches the full inference performance after a few queries.

### Data

We evaluate our approach on three empirical temporal networks. The first network represents reported pig movements in Switzerland, and we abbreviate it as PIG. We assume that a disease can only spread from the holding of origin to the holding of arrival and not the other way around and hence the network is directed. We consider a time period from January 1, 2015 to December 31, 2017 and the resulting network contains 8176 nodes and 149, 960 timestamped edges with a daily time resolution.

The second network represents sexual contacts between sex workers and their clients and was introduced by Rocha et al.^30,31^. We abbreviate it as the ESCORT network. It is an undirected and bipartite network with a daily time resolution and, as in previous research^18,31^, we discard the first 1000 days of the data. The resulting network has 14,783 nodes and 43,906 timestamped edges and spans over 1, 232 days (roughly 3.4 years).

The third network represents face-to-face contacts, measured by proximity sensors, between 86 residents of a village in Malawi and has been introduced by Ozella et al.^32^. We abbreviate this network as the MALAWI network. The original data has a time resolution of 20 s, which is too granular for our purposes. Hence, we aggregated the data to get an hourly time resolution. An edge between two residents exists if they had at least two encounters (measured in 20 s increments) during a given hour. The resulting (undirected) network has 86 nodes and 5, 854 timestamped edges and spans over 321 h (roughly 13 days).

### Example

**Figure 1:**
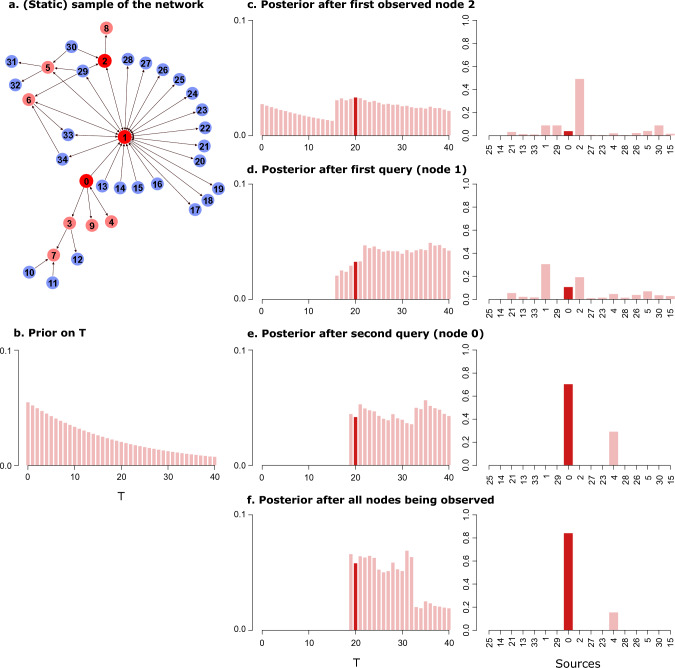
Example of our inference-querying cycle applied to one outbreak on the PIG network. (**a**) Static sample of the PIG network showing all infected nodes (in red) and all nodes with incoming and/or outgoing edges to infected nodes (in blue). The node labels of the infected nodes represent the order in which the nodes were infected. Thus, node $0$ is the true source of this outbreak it first infects node $1$. (**b**) Truncated geometric prior distribution on the duration until first observation *T*. (**c**) Marginal posterior distributions for *T* and sources *Q* after the initial observation of node $2$. The true source and true *T* are shown in darker red. (**d**) Marginal posterior distributions for *T* and *Q* after the first query decided by AKLD (node $1$). (**e**) Marginal posterior distributions for *T* and sources *Q* after the second query (node $0$). (**f**) Marginal posterior distributions for *T* and sources *Q* after all nodes in the network have been observed.

Figure 1 shows how our approach, more specifically the AKLD querying strategy, applies to one outbreak on the PIG network. Panel (c) shows that after initially observing node $2$ as infected the marginal source posterior deems node $2$ the most likely source. AKLD then decides to query the very central node $1$ as the possible sources seem to disagree most about its state. Node $1$ turns out to be infected and becomes the most likely source (Panel (d)). Note how this renders node $29$ an impossible source with a zero posterior probability. This is due to node $29$ only having one (static) path to the infected node $1$ via node $2$. However, it is not a time-respecting path (not shown in the Figure) and thus not a possible transmission pathway. AKLD next queries the true source node $0$ which leaves only two possible sources, the nodes $0$ and $4$ (Panel (e)). Note how in this example two queries are enough to become very confident about determining the source node. In fact, the final posterior after observing all nodes’ states in the network (Panel (f)) is not substantially different from the posterior after two queried nodes.

### Source inference with fully observed set of nodes

The first set of results is concerned with inferring the source of an epidemic spread based on the fully observed set of nodes $O_{t_1}=V$. For this, we conducted different sets of 500 experiments for the three empirical networks introduced above, involving different durations until the first observation *T* and different epidemic settings. Each experiment consists of (1) a simulation of a ground truth outbreak starting from a source node and starting time picked uniformly at random and restricted to have a final outbreak size of at least 10 nodes at the time of inference $t_0 + T$, and (2) source inference based on the fully observed set of nodes. For the PIG and MALAWI network, we set the epidemic parameters $\beta$ and $\mu$ such that $R_0 \approx 1.5$ and $R_0 \approx 2$. For the ESCORT network, we replicate the experimental setup in Antulov-Fantulin et al.^18^ and set $\beta =0.3$ and $\mu =0.01$.

For the PIG and MALAWI network we use a geometric prior on *T* with mean equal to the true value of *T*, truncated at twice the value of the true *T*. For the ESCORT network, we again keep with Antulov-Fantulin et al.^18^ and put a uniform prior on possible starting times in the interval $[t_0 - 25,\; t_0 + 25]$. The first step of our approach consists of computing the individual node state probabilities based on simulating outbreak scenarios from each possible source *Q* and each *T*. We use $n=10{,}000$ simulations for each (*Q*, *T*) pair. The average CPU time consumed by this simulation process is shown in Supplementary Fig. S16.

The key output of our inference approach is the bivariate posterior in Eq. (1) that we can marginalize to get the marginal source posterior. We use this marginal posterior distribution to compute three different measures that allow us to evaluate the source inference performance. First of all, we compute the rank of the true source $q_0$ in the sorted and ranked source posterior distribution. We then average the ranks of the true source over all experiments to get the *average rank*. Second, we compute the *precision* which is simply the fraction of experiments for which the true source has rank 1. Finally, we compute the 95% *credible set size* (CSS)^26,39^, which, in our case, is simply the size of the smallest set of nodes that have a cumulative posterior probability of at least 95%.

**Figure 2:**
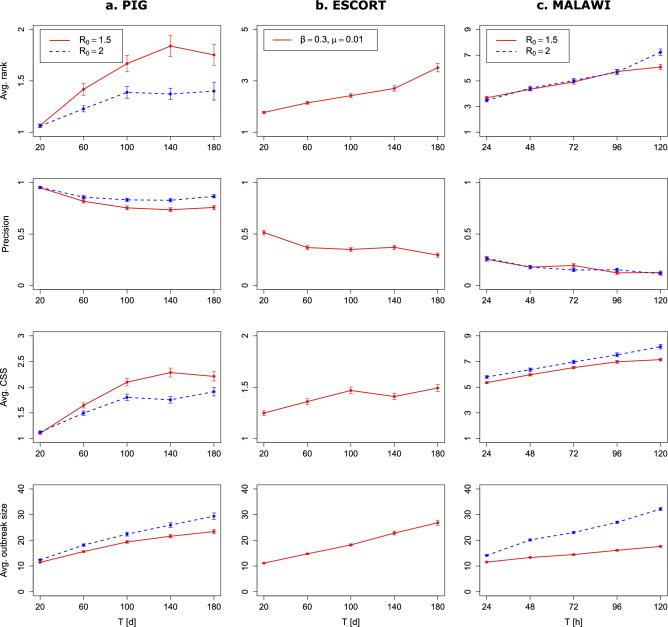
The source (mean-field-like) inference results for different durations until the first observation *T* if the states of all nodes are observed. (**a**) Source inference results on the PIG network for two different sets of experiments, one with a basic reproduction number $R_0=1.5$ and one with $R_0=2$. (**b**) Source inference results on the ESCORT network for infection parameters $\beta = 0.3$ and $\mu =0.01$, analogous to the setup in Antulov-Fantulin et al.^18^. (**c**) Source inference results on the MALAWI network for two different sets of experiments, one with a basic reproduction number $R_0=1.5$ and one with $R_0=2$. For all three networks, we measure the inference performance with three different measures, namely the average rank, the precision, and the average credible set size (CSS). In addition, we show the average outbreak size. Every point on a curve represents an average of 500 experiments. Error bars represent ± one standard error (s.e.m.), but errors are, in some cases, small and hardly discernible.

The results are shown in Fig. 2. Unsurprisingly, all three performance measures indicate that source inference becomes harder for larger *T*. In other words, the longer an outbreak has time to evolve, the harder it becomes to infer which node started the outbreak. Moreover, it is substantially easier to infer the true source node on the PIG network as compared to the ESCORT and, in particular, the MALAWI network. A closer look at the results for the PIG network ($R_0=1.5$) reveals that at $T=20$, 77.4% of the experiments have only one source node left as a possible source after all node states are observed (not shown in Figure). This fraction decreases to 48.4% at $T=180$. It becomes evident that it is fairly easy to infer the true source when all nodes can be observed. This can be partially explained by the PIG network being directed, which leads to strong topological constraints on possible transmission pathways. Furthermore, a comparison of the (static) densities of the networks reveals that the density of the MALAWI network (0.08) is two orders of magnitudes larger than the density of the PIG (0.0007) and the ESCORT (0.0003) network. In the Supplementary Information, we provide further source inference results of our approach when applied to well-known static network models and show that higher densities lead to a substantially worse source inference performance.

A somewhat counterintuitive result is that it seems to be easier to infer the source on the PIG network for $R_0=2$ as compared to $R_0=1.5$. A possible explanation for this is that outbreaks on the PIG network do not evolve past a relatively small and local scale. From Fig. 2, we can for example see that an outbreak size larger than 30 is unlikely, which is extremely small compared to the size of the network. At this scale, it may actually be beneficial for source inference to observe more infected nodes. This finding is consistent with that of Antulov-Fantulin et al.^18^, who show that on 4-connected lattice graphs, source detectability is higher for large transmission probabilities.

### Active querying

Having established the full inference results as a benchmark, we now present the active querying results. The experimental setup is similar to the previous section. After generating a ground truth outbreak with the same specifications as above, we conduct the inference and querying process at time $t_1$. The process starts with picking one node uniformly at random, as the first observed node, from all activated nodes. Then, the inference and querying are performed iteratively up to a specified number of queries. For the PIG and ESCORT networks, we perform 30 queries in each experiment, while for the MALAWI network, we continue the querying until each node is observed (85 queries). Again, we use the average rank, the precision, and the average CSS to measure the source inference performance at each step of the querying process. The average CPU time consumed by the different querying strategies is shown in Supplementary Fig. S16. It is evident that the simulation process is substantially more expensive than the querying and inference process. Moreover, AKLD is one order of magnitude more expensive than the other querying strategies.

**Figure 3:**
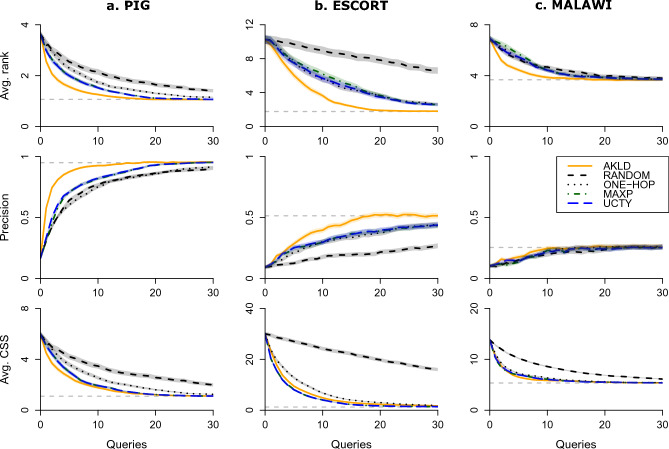
Querying curves for all five strategies, where the horizontal dashed lines represent the source inference performance when all node states have been observed. (**a**) Average rank, precision, and average CSS at different stages of the querying process on the PIG network for $T=20$ and $R_0=1.5$. (**b**) Results for the ESCORT network for $T=20$ and epidemic parameters $\beta =0.3$ and $\mu =0.01$. (**c**) Results for the MALAWI network for $T=24$ and $R_0=1.5$. To improve visibility and comparability, we only plot the curves up to 30 queries even though we performed a total of 85 queries on the MALAWI network. All curves contain error areas that represent ± one standard error (s.e.m.).

First, we are interested in comparing the performance of the five querying strategies. Figure 3 shows the querying curves for all five strategies for small values of *T*. In order to relate the querying results to the full inference performance, we always indicate the full inference performance with horizontal dashed lines. It is apparent from this Figure that AKLD is the dominant strategy, rarely performing worse than the other strategies. On the contrary, AKLD often leads to a substantial improvement even when compared to the other two active strategies MAXP and UCTY. Therefore, we will focus on the AKLD strategy in the remainder of this section. The results for all five strategies are shown in Supplementary Figs. S13–S15.

**Figure 4:**
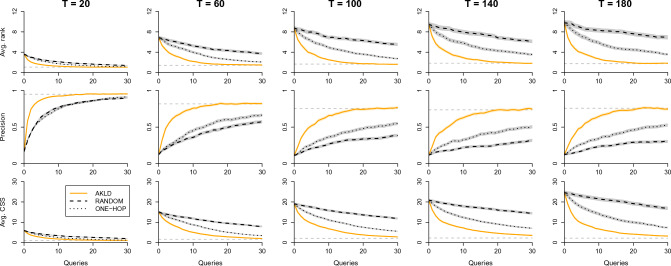
The AKLD strategy as compared to the baseline querying strategies for the PIG network and $R_0=1.5$. The first, second, and third row of plots show the average rank, precision, and average CSS, respectively, for different *T*. The horizontal dashed lines indicate the full inference performance. All curves contain error areas that represent ± one standard error (s.e.m.).

Second, we conduct an extensive comparison of AKLD with the two baseline strategies ONE-HOP and RANDOM for all three networks. Figure 4 presents the results for the PIG network. The results show a remarkable difference between AKLD and the baseline methods, even for large *T*. There are two other interesting effects. First, the relatively small and sparse outbreaks seem to favor the ONE-HOP baseline over the RANDOM strategy. Second, while the two baseline strategies seem to deteriorate over time (curves increasingly end further from the full inference performance), AKLD does not seem to suffer strongly from this effect and still gets close to the full inference performance at $T=180$ after only 30 queries.

**Figure 5:**
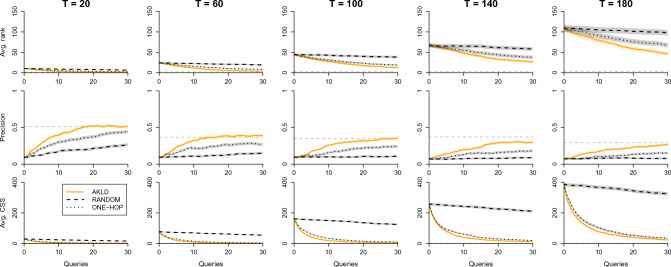
The AKLD strategy as compared to the baseline querying strategies for the ESCORT network and epidemic parameters $\beta =0.3$ and $\mu =0.01$. The first, second, and third row of plots show the average rank, precision, and average CSS, respectively, for different *T*. The horizontal dashed lines indicate the full inference performance. All curves contain error areas that represent ± one standard error (s.e.m.).

Figure 5 presents the results for the ESCORT network. Here too, the AKLD strategy can lead to substantial improvements as compared to the baselines, especially when the precision is considered. Note that the precision is considerably more robust to outliers than the average rank. That is why the precision curves still approach the full inference performance while the average rank curves are far from the full inference performance for later *T*. It can also be seen that the curves for the RANDOM strategy are almost flat, especially for later *T*. The reason for this is that on this network there are many experiments with a large number of possible sources and therefore the RANDOM strategy typically samples from a large pool of nodes, of which many are not especially informative. Finally, we see that for later *T* we would need to query more than 30 nodes for all three strategies to reach the full inference performance.

**Figure 6:**
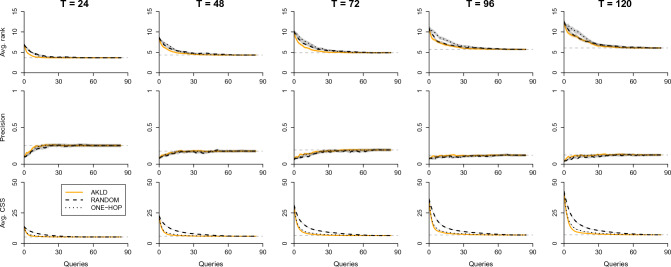
The AKLD strategy as compared to the baseline querying strategies for the MALAWI network and $R_0=1.5$. The first, second, and third row of plots show the average rank, precision, and average CSS, respectively, for different *T*. The horizontal dashed lines indicate the full inference performance. All curves contain error areas that represent ± one standard error (s.e.m.).

Figure 6 presents the results for the MALAWI network. First of all, we note that source inference is substantially harder than in the other two cases, a result we already observed and discussed in the previous section. Second, while AKLD still seems to be the best strategy overall, its benefit as compared to the other strategies, especially ONE-HOP, is marginal. Finally, while we proceed here with the querying until all nodes have been queried and observed, it is evident from the Figure that querying roughly a third of all nodes is in most cases enough to reach the full inference performance.

We provide further results in the Supplementary Information. First of all, a comparison of the AKLD querying curves for $R_0=1.5$ with those for $R_0=2$ can be found in Supplementary Figs. S17 and S18. As in the full inference results, a higher $R_0$ leads to better querying results for the PIG network. By contrast, a higher $R_0$ leads to a slight deterioration of the querying results on the MALAWI network. Second, we test the effect of *T* being uncertain as compared to *T* being known for the PIG network. As expected, knowing the true value of *T* leads to slightly improved source inference results as compared to *T* being unknown (see Supplementary Fig. S19). Finally, the Supplementary Information also provides an extensive evaluation of the querying approach on static network models.

## Discussion

The main objective of this paper is to pose and study an active learning problem in the context of source inference in epidemics on contact networks, namely the active querying problem. The idea is to query the state of unobserved nodes, starting from a set of one or only a few observed nodes, in such a way as to learn as much as possible about the source of the outbreak with as few queries as possible. We propose three active querying strategies, two of them based on information-theoretic principles, and compare them to simple baseline querying strategies. The most striking result to emerge from the analysis is that the AKLD strategy, which selects the node with the maximum average KL-divergence between individual node state predictions and the consensus prediction, is overall the dominant strategy and shows promising results on two out of the three empirical networks we analyzed. The AKLD strategy can be interpreted as a *query-by-committee* learning strategy^28^, where the committee is the set of possible source-duration pairs. Effectively, the strategy queries controversial nodes and may thus be able to narrow down the search for the true source more quickly, on average, than the other strategies.

The active querying problem setting we define, as well as the approach we propose to solve the active querying problem, attempt to be as general as possible and close to a practical application. First of all, we assume that we do *not* have access to full snapshots of the epidemic states of a substantial fraction (or all) of the nodes, an assumption often made by related work^14,18^. We also do not assume that infection times are observed or that the starting time of the outbreak is known. Second, our approach is general enough to be applied to either static or temporal, as well as undirected or directed contact networks. Further generalizations, for example to weighted networks, are relatively straightforward. While the main text focuses on the application to temporal contact networks, we also provide extensive results on static network models in the Supplementary Material. Third, we present a Bayesian inference mechanism where non-trivial prior distributions can be used to model prior knowledge about possible sources and the duration of the epidemic. To make the inference tractable, we use a node independence assumption similar to the independence assumption made by Naive Bayes. This independence assumption is common in the source inference literature and has been shown to work well for source detection^13,14^. Key to both our inference and the querying procedure is the ability to compute individual node state probabilities given a source and duration. Here, we make use of efficient event-driven large-scale Monte-Carlo simulations instead of deterministic message-passing algorithms to avoid biased probability estimates due to the node independence assumption used for such deterministic approaches^13,14^. The Monte-Carlo procedure, even though computationally much more expensive than deterministic methods, can often be run in a reasonable time for practical purposes. A convenient side effect of this is that Monte-Carlo simulations are model-agnostic and, thus, any propagation model that can be simulated efficiently could be used. Both the simulation approach and AKLD have a time complexity containing a factor that is quadratic in *N*. Hence, our approach may be impractical for really large networks. In this case, parallelizing the simulation procedure is straightforward or, as an alternative, we could use a deterministic approach such as IBA for temporal networks^14^ with a substantially smaller overall time complexity ($\mathcal {O}(N \cdot C)$) than Monte-Carlo simulations. For the querying, we can resort to the UCTY or MAXP strategy which have a time complexity that is linear in *N*.

A number of limitations to this study are worth pointing out. One weakness, apart from the mean-field-like independence assumption in modeling the likelihood, is that we assume the epidemic model and its parameters to be known. Further work is required to establish solutions that deal with a fully uncertain setting, be it model-agnostic approaches or a Bayesian solution that puts a prior on a broad range of possible epidemic models. One promising avenue could be to pair our querying idea with Approximate Bayesian Computation (ABC), in which the likelihood for all unknown parameters would be estimated by means of a rejection sampling process that rejects all samples that are too dissimilar to the observed data, and which has already been applied in an epidemic context on static networks^37^. An additional limitation is our focus on outbreaks caused by a single source node instead of possibly multiple sources. As has been noted by Lokhov et al.^13^, future research could investigate Monte-Carlo search procedures to find likely sets of source nodes. Finally, the active querying problem that we have introduced deserves closer investigation from a theoretical point of view, with the aim of better understanding the theoretical limits of active querying and why and under what conditions certain active querying strategies work well. It could also be interesting to further investigate the topological properties of nodes that are queried early on by active strategies.

The findings of this study have a number of practical implications. Unsurprisingly, the identification of patient zero works best in the early stages of an epidemic and on sparse networks, such as the PIG network, where the topology, especially the directed nature of the network, puts many constraints on possible transmission pathways. Our study demonstrates how contact networks can be used in conjunction with appropriately gauged parametric spreading models to make more informed decisions in a contact tracing process by recommending which nodes to query. The proposed AKLD query strategy in some cases leads to a dramatic improvement in the inference performance as compared to other strategies and often allows us to infer the source node after just a few queries and with the same confidence as if all nodes had been observed.

Recent events have shown that tracing the source of infections and possible nodes at risk is an important element for understanding (and possibly containing) epidemics. Given that in practice ascertaining the infection status of a given node is costly—be it in terms of finding the individual at a given time, running a test, or societally due to privacy considerations—testing all nodes seems impractical. Hence, we believe that active querying approaches could pave the way for a more practical and cost-efficient approach to identifying the source of an epidemic that allocates the tracing resources to the most informative nodes.

## Supplementary Information

Supplementary Information.

## Data Availability

The Swiss pig movement data are collected by Identitas AG. For research purposes, a data request can be sent to Identitas AG, Stauffacherstrasse 130A, 3014 Bern, Switzerland (https://www.identitas.ch/). The network of sexual contacts between sex workers and their clients can be downloaded from the Supporting Information of the article by Rocha et al.^31^. Finally, the network of contacts between residents of a village in Malawi is described in Ozella et al.^32^ and can be downloaded from SocioPatterns (http://www.sociopatterns.org/datasets/).
