# Supplementary material for: Active querying approach to epidemic source detection on contact networks
Source: Sci Rep. 2023 Jul 13;13:11363. doi: 10.1038/s41598-023-38282-8 (PMC10345105; doi:10.1038/s41598-023-38282-8)
Supplement: Supplementary file 1 — Supplementary Information. [file 41598_2023_38282_MOESM1_ESM.pdf]

Supplementary Information for

Active Querying Approach to Epidemic Source Detection on Contact Networks

Martin Sterchi, Lorenz Hilfiker, Rolf Grütter, Abraham Bernstein

Martin Sterchi.
E-mail: martin.sterchi@fhnw.ch

Log-Sum-Exp Trick

As mentioned in the main text, we aim to compute the following posterior distribution:

$$P(Q,T\,|\,E_{t_1}) = \frac{P(E_{t_1}\,|\,Q,T)\cdot P(T)}{\sum_{Q'}\sum_{T'} P(E_{t_1}\,|\,Q',T')\cdot P(T')} \tag{1}$$

In order to avoid numerical underflow, we apply the log-sum-exp trick (see for example p. 86 in Murphy (1)). If we take the (natural) logarithm on both sides of equation [1], we get

$$\begin{aligned}\log\, P(Q,T\,|\,E_{t_1}) &= \log\, P(E_{t_1}\,|\,Q,T) + \log\, P(T) - \log\left[\sum_{Q'}\sum_{T'} P(E_{t_1}\,|\,Q',T')\cdot P(T')\right] \\ &= \log\, P(E_{t_1}\,|\,Q,T) + \log\, P(T) - \log\left[\sum_{Q'}\sum_{T'} \exp\left(\log\, P(E_{t_1}\,|\,Q',T') + \log\, P(T')\right)\right].\end{aligned} \tag{2}$$

The first step of the log-sum-exp trick is to compute $b_{q,d} = \log\, P(E_{t_1}\,|\,Q=q, T=d) + \log\, P(T=d)$ for all possible source-duration pairs (q,d). The second step consists of finding $B = \max_{q,d}\, b_{q,d}$. With this we can rewrite the second part of the expression in [2] as follows:

$$\begin{aligned}\log\left[\sum_{Q'}\sum_{T'} \exp\left(\log\, P(E_{t_1}\,|\,Q',T') + \log\, P(T')\right)\right] &= \log\left[\sum_{q\in V}\sum_{d\in\mathcal{T}} \exp\left(b_{q,d}\right)\right] \\ &= \log\left[\exp\left(B\right)\cdot\left(\sum_{q\in V}\sum_{d\in\mathcal{T}} \exp\left(b_{q,d}-B\right)\right)\right] \\ &= B + \log\left[\sum_{q\in V}\sum_{d\in\mathcal{T}} \exp\left(b_{q,d}-B\right)\right]\end{aligned} \tag{3}$$

The third step is to compute $\exp\left(b_{q,d}-B\right)$, which is much less prone to underflow, for all possible source-duration pairs (q,d). With this we can compute the last expression in [3]. Finally, we can plug everything into the expression in [2] and then compute $\exp\left(\log\, P(Q,T\,|\,E_{t_1})\right)$ to get the posterior.

Node State Probabilities

Here we briefly review two approaches to computing the individual node state probabilities $P_{q,d}(X_v(t))$ and then compare these two approaches to the approach of using Monte-Carlo simulations. The two approaches we review here are the *individual-based* and *pair-based approximation* (IBA and PBA, respectively), which have been introduced for static networks in previous research (2–4). The generalization to discrete-time temporal networks is straightforward (5), which is why we will focus on the static case here. IBA and PBA are approximate methods that, assuming an underlying SIR spreading process, deterministically model the time evolution of state probabilities for individual nodes (and additionally for pairs of nodes in the case of PBA). Specifically, the evolution of these state probabilities is described by $2N$ differential equations (in the case of IBA), one for every node and every possible state of the node. (Since the probabilities of states S, I, and R sum up to 1 for each node, the equations for the recovered state are redundant.) Note that IBA constitutes a computationally much cheaper alternative to the approach of using Monte-Carlo simulations and may thus be useful for applying our approach to very large networks.

Individual-Based Approximation. Defining the set of neighbors of a node v as $N_v = \{u;\ (v,u)\in E\}$, we can write down the exact evolution of the node state probabilities of a node v with the following two differential equations (4):

$$\frac{dP_{q,d}(X_v(t)=S)}{dt} = -\beta \sum_{u\in N_v} P_{q,d}(X_v(t)=S,\ X_u(t)=I) \tag{4}$$

$$\frac{dP_{q,d}(X_v(t)=I)}{dt} = \beta \sum_{u\in N_v} P_{q,d}(X_v(t)=S,\ X_u(t)=I) - \mu\cdot P_{q,d}(X_v(t)=I) \tag{5}$$

As we can see, both equations depend on the joint probability $P_{q,d}(X_v(t)=S, X_u(t)=I)$ and thus, we need to write down a differential equation for this probability as well:

$$\begin{aligned}\frac{dP_{q,d}(X_v(t)=S,X_u(t)=I)}{dt} &= \beta \sum_{w\in N_u\setminus v} P_{q,d}(X_v(t)=S, X_u(t)=S, X_w(t)=I) \\ &\quad -\beta \sum_{w\in N_v\setminus u} P_{q,d}(X_v(t)=S, X_u(t)=I, X_w(t)=I) \\ &\quad -\beta\cdot P_{q,d}(X_v(t)=S, X_u(t)=I) \\ &\quad -\mu\cdot P_{q,d}(X_v(t)=S, X_u(t)=I)\end{aligned} \tag{6}$$

The first term on the right side of the equation constitutes an in-flux due to the possibility that some third node w infects u. The second term is an out-flux due to the possibility that w infects v. The last two terms are out-fluxes due to the possibility of u infecting v and node u recovering, respectively. Importantly, this equation now depends on probabilities involving three nodes, which would require us to write down differential equations for those expressions. This cascade continues until we would have equations for probabilities involving all nodes in the network. Such a system has typically too many equations and it cannot be solved in a tractable way (4).

The simplest way to break this cascade is to assume that $P_{q,d}(X_v(t), X_u(t)) = P_{q,d}(X_v(t)) \cdot P_{q,d}(X_u(t))$, i.e., assuming independence between neighboring nodes v and u. Equations [4] and [5] can then be simplified to:

$$\frac{dP_{q,d}(X_v(t) = S)}{dt} = -\beta \cdot P_{q,d}(X_v(t) = S) \sum_{u \in N_v} P_{q,d}(X_u(t) = I) \tag{7}$$

$$\frac{dP_{q,d}(X_v(t) = I)}{dt} = \beta \cdot P_{q,d}(X_v(t) = S) \sum_{u \in N_v} P_{q,d}(X_u(t) = I) - \mu \cdot P_{q,d}(X_v(t) = I) \tag{8}$$

This is a closed (but approximate) system of $2N$ differential equations and can be solved numerically. It is called individual-based approximation (IBA). Note how the complexity is reduced to linear growth in N, as compared to exponential growth in N for the continuous-time Markov chain (3).

Note that IBA tends to underestimate a node's probability of being susceptible, and overestimate its probability of being infectious or recovered (3). This can be seen from the fact that $P_{q,d}(X_v = S \mid X_u = I) \leq P_{q,d}(X_v = S)$ must always hold, and, consequently, $P_{q,d}(X_v = S,\, X_u = I) = P_{q,d}(X_v = S \mid X_u = I) \cdot P_{q,d}(X_u = I) \leq P_{q,d}(X_v = S) \cdot P(X_u = I)$.

Pair-Based Approximation. In many cases, the independence assumption at the level of nodes will be too strong a simplification. We can achieve a better approximation by assuming independence at the level of pairs. This approach is called the pair-based approximation (PBA). The price to pay for taking pair-based interactions into account is an increased number of equations to close the system: PBA requires equations [4] and [5], as well as three equations for each edge, which amounts to $2N + 3|E|$ equations in total.

PBA closes the system at the level of triples (instead of at the level of pairs). For this, we need to express the joint probability $P_{q,d}(X_v(t), X_u(t), X_w(t))$ in equation [6] in terms of probabilities for pairs of nodes and single nodes. Based on the chain rule of probability, we can rewrite the joint probability as $P_{q,d}(X_v(t), X_u(t), X_w(t)) = P_{q,d}(X_v(t) \mid X_u(t), X_w(t)) \cdot P_{q,d}(X_w(t) \mid X_u(t)) \cdot P_{q,d}(X_u(t))$. If we assume that $X_v(t)$ is conditionally independent of $X_w(t)$ given $X_u(t)$, we can rewrite the joint probability, using $P_{q,d}(X_v(t) \mid X_u(t), X_w(t)) = P_{q,d}(X_v(t) \mid X_u(t))$:

$$\begin{aligned} P_{q,d}(X_v(t), X_u(t), X_w(t)) &= P_{q,d}(X_v(t) \mid X_u(t)) \cdot P_{q,d}(X_w(t) \mid X_u(t)) \cdot P_{q,d}(X_u(t)) \\ &= \frac{P_{q,d}(X_v(t), X_u(t))}{P_{q,d}(X_u(t))} \cdot \frac{P_{q,d}(X_w(t), X_u(t))}{P_{q,d}(X_u(t))} \cdot P_{q,d}(X_u(t)) \\ &= \frac{P_{q,d}(X_v(t), X_u(t)) \cdot P_{q,d}(X_w(t), X_u(t))}{P_{q,d}(X_u(t))}. \end{aligned} \tag{9}$$

Now, the joint probability $P_{q,d}(X_v(t), X_u(t), X_w(t))$ has been expressed in terms of simpler constructs. Note that this will be exact on trees since $X_v(t)$ will always be independent of any upstream nodes $X_w(t)$ given its parent $X_u(t)$. A more general approximation that applies to so-called closed triples is as follows (2, 4):

$$P_{q,d}(X_v(t), X_u(t), X_w(t)) = \frac{P_{q,d}(X_v(t), X_u(t)) \cdot P_{q,d}(X_u(t), X_w(t)) \cdot P_{q,d}(X_w(t), X_v(t))}{P_{q,d}(X_v(t)) \cdot P_{q,d}(X_u(t)) \cdot P_{q,d}(X_w(t))} \tag{10}$$

Note, however, that this form of approximation requires more equations to be solved than the one in equation [9] and becomes even less practical for graphs of non-trivial size.

With the closure in equation [9], the set of differential equations needed for PBA are equations [4] and [5], as well as:

$$\begin{aligned} \frac{dP_{q,d}(X_v(t) = S, X_u(t) = I)}{dt} &= \beta \sum_{w \in N_u \setminus v} \frac{P_{q,d}(X_v(t) = S, X_u(t) = S) \cdot P_{q,d}(X_u(t) = S, X_w(t) = I)}{P_{q,d}(X_u(t) = S)} \\ &\quad - \beta \sum_{w \in N_v \setminus u} \frac{P_{q,d}(X_v(t) = S, X_u(t) = I) \cdot P_{q,d}(X_v(t) = S, X_w(t) = I)}{P_{q,d}(X_v(t) = S)} \\ &\quad - \beta \cdot P_{q,d}(X_v(t) = S, X_u(t) = I) \\ &\quad - \mu \cdot P_{q,d}(X_v(t) = S, X_u(t) = I) \end{aligned} \tag{11}$$

$$\begin{aligned} \frac{dP_{q,d}(X_v(t) = S, X_u(t) = S)}{dt} &= -\beta \sum_{w \in N_v \setminus u} \frac{P_{q,d}(X_v(t) = S, X_u(t) = S) \cdot P_{q,d}(X_v(t) = S, X_w(t) = I)}{P_{q,d}(X_v(t) = S)} \\ &\quad - \beta \sum_{w \in N_u \setminus v} \frac{P_{q,d}(X_v(t) = S, X_u(t) = S) \cdot P_{q,d}(X_u(t) = S, X_w(t) = I)}{P_{q,d}(X_u(t) = S)} \end{aligned} \tag{12}$$

Equation [12] is required because the probability of nodes v and u both being in the S-state appears in [11]. The two terms on the right side of equation [12] determine the out-flux due to an infection of node v and u by some third node w, respectively. PBA leads to $2N + 3|E|$ equations in total. Hence, every edge in the network requires three differential equations, which may seem a bit counterintuitive, as we may expect only two since we have two equations for pairs ([11] and [12]). However, we need separate equations for $P_{q,d}(X_v(t) = S, X_u(t) = I)$ and for $P_{q,d}(X_v(t) = I, X_u(t) = S)$.

Comparison of Methods. Considering that previous works (5, 6) have used IBA or similar methods for source inference, we now briefly examine the quality of the node state probability approximations IBA and PBA with respect to MCS. More specifically, we compare the approximate node state probabilities to the approximately correct node state probabilities resulting from MCS for all nodes in the network and at different times during the epidemic. The analysis is performed on Erdős-Rényi (ER) networks with varying density and on a 4-connected lattice. All networks are of size $N = 100$.

For each possible source node, we run IBA, PBA, and MCS with $n = 10{,}000$, using Kiss et al.'s implementation of IBA and PBA, which can be found here (7). As for the epidemic parameters, we set $\mu = 1$ (without loss of generality) and determine the transmission rate β such that we achieve a basic reproduction number of $R_0 \approx 2$.

The individual node state probabilities resulting from IBA and PBA are compared to those from MCS via the cross-entropy loss (sometimes called log loss) which is one way to measure the difference between discrete probability distributions. We use it to measure how much the approximate node state probabilities (given a source node) deviate from the node state probabilities that result from the Monte-Carlo simulations. Here, we define the cross-entropy loss for IBA, although it could be defined in a similar way for PBA. Note that the loss is measured for a given source q and duration d, and at some time t:

$$J(q,d,t) = -\frac{1}{N} \sum_{v \in V} \sum_{x \in \{S,I,R\}} P_{q,d}^{MCS}(X_v(t) = x) \cdot \log(\, P_{q,d}^{IBA}(X_v(t) = x)\,) \qquad [13]$$

The cross-entropy loss allows us to aggregate the differences in the three node state probabilities over all nodes in the network into one single loss.

Figure S1 shows the average cross-entropy loss over time and for four different networks. As expected, the average cross-entropy loss is consistently lower for PBA than for IBA. A similar result is found for aggregate measures of epidemics, such as the expected prevalence, where PBA provides more precise results than IBA (4). Another interesting aspect is that increasing the average degree, and thus the density of ER networks, seems to decrease the loss of IBA considerably with respect to the loss of PBA, which can be explained by the node independence assumption being more justified in strongly connected networks (2).

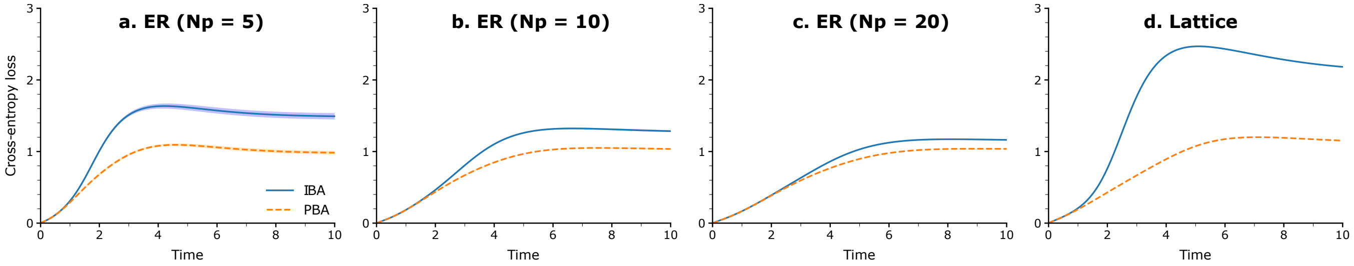

Fig. S1. Average cross-entropy loss of IBA and PBA with respect to MCS (averaged over 100 source nodes). (a–c) Average loss for Erdős-Rényi (ER) networks with $N = 100$ nodes and average degree 5, 10, and 20, respectively. (d) Average loss for a 4-connected lattice (with periodic boundary conditions) of size $N = 10 \times 10$. Error areas indicate $\pm$ one standard error (s.e.m.) but are hardly discernible. In the case of the lattice, this can be explained by the fact that the topology will be identical for each source node and, as a result, only a negligible amount of variance in the loss (due to small variations in the MCS node state probabilities) remains.

While IBA and PBA are at an obvious disadvantage compared to MCS with regard to accuracy, their computational cost is considerably lower than for MCS. For example, the average CPU time for running IBA for one source on the ER network with $Np = 5$ is 0.0763 seconds, 95% CI [0.0752, 0.0773]. For PBA the average CPU time is 2.71 seconds, 95% CI [2.69, 2.74], and for MCS ($n = 10{,}000$) the average CPU time is 89.33 seconds, 95% CI [88.50, 90.15].

Toy Example on Static Network

Source Inference. Figure S2 illustrates the source inference problem with a small static network and a simple spreading process, where nodes never recover, i.e., $\mu = 0$, and T is known. Panel (b) shows the exact and approximate (mean-field-like) source posterior distribution for a scenario in which all four nodes are observed as infectious. While the two distributions qualitatively agree that node 1 is the most likely source node, they differ substantially in the posterior probabilities for sources 0 and 1. The reason is that the independence assumption leads to an underestimation of the true likelihoods, especially for sources 0, 2, and 3. The likelihood for source 1 is underestimated the least, which translates to node 1 having too strong of a weight in the computation of the (approximate) posterior distribution.

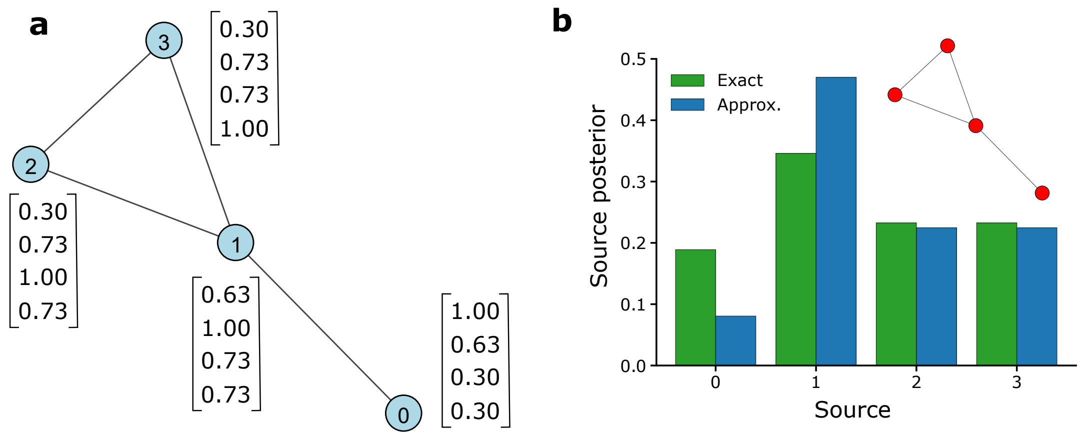

Fig. S2. A toy example that demonstrates the source inference problem. (a) A simple static network with four nodes. The vectors assigned to each node contain the exact node state probabilities of being infectious at $t^* = 10$ (for all four sources) given a simple *susceptible-infectious* (SI) spreading process with transmission rate $\beta = 0.1$ (no recovery). For instance, node 0 has a probability of 1.0 to be infectious given it is the source itself, a probability of 0.63 given that node 1 is the source, and a probability of 0.3 given that either node 2 or 3 is the source. (b) Exact and approximate (mean-field-like) source posterior distribution if at $t^* = 10$ all four nodes are observed as infectious. The exact solution is based on a continuous-time Markov chain that was solved by numerical integration (see Supplementary Information).

Active Querying. We illustrate the active querying problem based on the AKLD strategy in Figure S3. After initially observing node 3 to be infectious, AKLD queries node 0, which may initially seem counterintuitive as one might expect node 1 to be queried first. However, due to its centrality, node 1 is likely to be infected by all sources and thus there will not be much disagreement among the sources about the state of node 1. After having observed the state of all nodes, the nodes 2 and 3 are equally likely to be the source because of the symmetry in the network. In the toy example, we use the mean-field-like independence assumption to compute the posteriors. One major problem with this assumption can be demonstrated if we imagine that node 1 is queried first (instead of 0) and turns up susceptible. In that case, node 0 would continue to be a possible source even though its path to the first observed node 3 is blocked by a susceptible node (1).

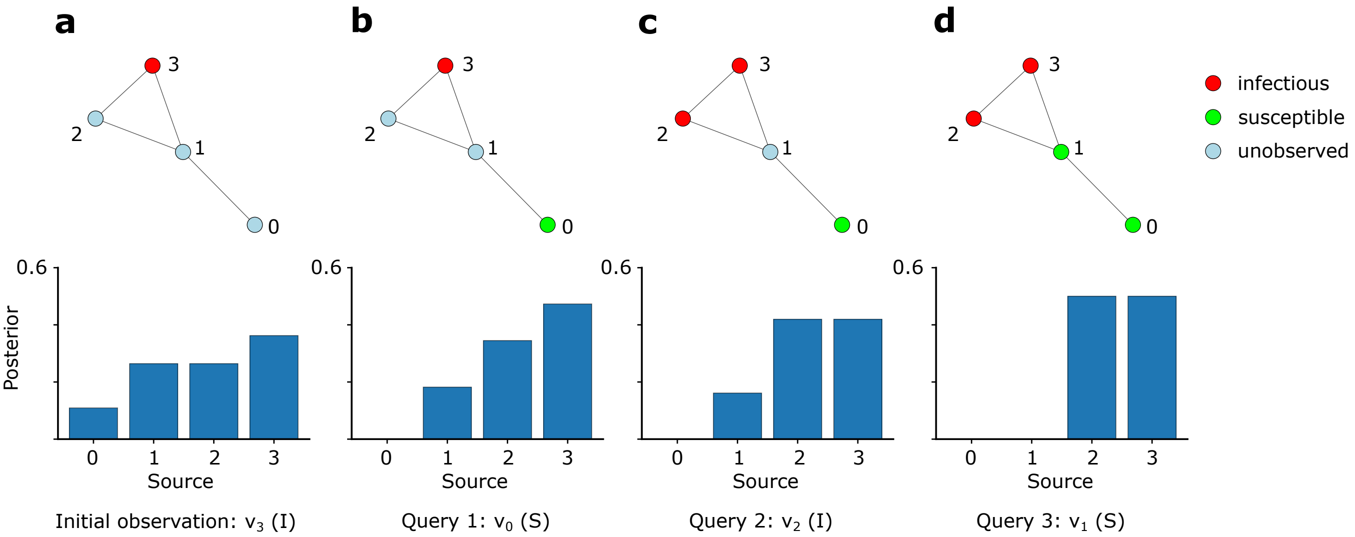

Fig. S3. A toy example that demonstrates the AKLD strategy on a static network. (a) Initially, only node 3 is observed as infectious. (b–d) The AKLD strategy then queries the nodes 0, 2, and 1 in this order. Their states are susceptible, infectious, and susceptible, respectively. The bar plots show the source posterior distributions at each step, which are computed based on the mean-field-like assumption.

Exact Solution of Toy Example. In the toy example, we consider a simple *susceptible-infectious* (SI) spreading model with transmission rate β and no recovery. There are a total of $2^4 = 16$ possible states. Figure S4 shows all states and the transitions that are possible for this example.

The system can be modeled as a continuous-time Markov chain. We denote as P_{ISSS} the probability that the system is in state $ISSS$, meaning that node 0 is in state I and all other nodes are in state S. Furthermore, $\dot{P}_{ISSS}$ denotes the time derivative of the probability of state $ISSS$. From Figure S4 we can see that the state $ISSS$ can only transition to one other state, namely $IISS$. It changes its state at rate β. The full set of master equations is as follows:

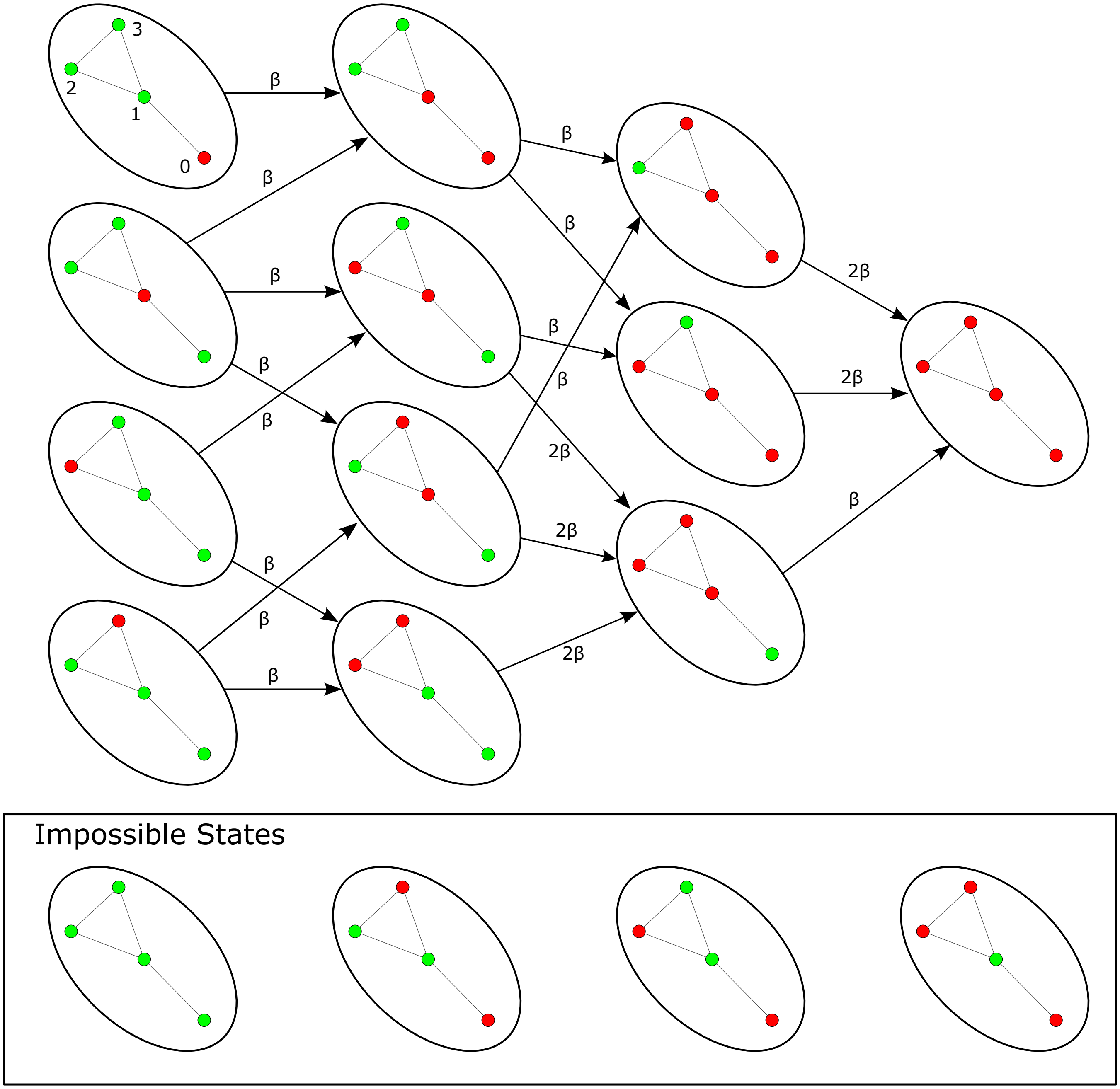

Fig. S4. The 16 possible states of the continuous-time Markov chain and the possible transmissions between states. Note that there are three states that are impossible due to the topology of the network. The state where all four nodes are susceptible is also not relevant here.

$$
\begin{aligned}
\dot{P}_{ISSS} &= -\beta \cdot P_{ISSS} \\
\dot{P}_{SISS} &= -3\beta \cdot P_{SISS} \\
\dot{P}_{SSIS} &= -2\beta \cdot P_{SSIS} \\
\dot{P}_{SSSI} &= -2\beta \cdot P_{SSSI} \\
\dot{P}_{IISS} &= \beta \cdot (P_{ISSS} + P_{SISS}) - 2\beta \cdot P_{IISS} \\
\dot{P}_{SIIS} &= \beta \cdot (P_{SISS} + P_{SSIS}) - 3\beta \cdot P_{SIIS} \\
\dot{P}_{SISI} &= \beta \cdot (P_{SISS} + P_{SSSI}) - 3\beta \cdot P_{SISI} \\
\dot{P}_{SSII} &= \beta \cdot (P_{SSIS} + P_{SSSI}) - 2\beta \cdot P_{SSII} \\
\dot{P}_{IISI} &= \beta \cdot (P_{IISS} + P_{SISI}) - 2\beta \cdot P_{IISI} \\
\dot{P}_{IIIS} &= \beta \cdot (P_{IISS} + P_{SIIS}) - 2\beta \cdot P_{IIIS} \\
\dot{P}_{SIII} &= 2\beta \cdot (P_{SIIS} + P_{SISI} + P_{SSII}) - \beta \cdot P_{SIII} \\
\dot{P}_{IIII} &= 2\beta \cdot (P_{IISI} + P_{IIIS}) + \beta \cdot P_{SIII}
\end{aligned}
$$

As outlined in Kiss et al. (4), the above master equations can be written in matrix form as

$$\dot{P} = AP, \tag{14}$$

where P corresponds to a column vector with the probabilities of each state (in the order of the master equations) and A is the following matrix:

$$A = \begin{pmatrix}
-\beta & 0 & 0 & 0 & 0 & 0 & 0 & 0 & 0 & 0 & 0 & 0 \\
0 & -3\beta & 0 & 0 & 0 & 0 & 0 & 0 & 0 & 0 & 0 & 0 \\
0 & 0 & -2\beta & 0 & 0 & 0 & 0 & 0 & 0 & 0 & 0 & 0 \\
0 & 0 & 0 & -2\beta & 0 & 0 & 0 & 0 & 0 & 0 & 0 & 0 \\
\beta & \beta & 0 & 0 & -2\beta & 0 & 0 & 0 & 0 & 0 & 0 & 0 \\
0 & \beta & \beta & 0 & 0 & -3\beta & 0 & 0 & 0 & 0 & 0 & 0 \\
0 & \beta & 0 & \beta & 0 & 0 & -3\beta & 0 & 0 & 0 & 0 & 0 \\
0 & 0 & \beta & \beta & 0 & 0 & 0 & -2\beta & 0 & 0 & 0 & 0 \\
0 & 0 & 0 & 0 & \beta & 0 & \beta & 0 & -2\beta & 0 & 0 & 0 \\
0 & 0 & 0 & 0 & \beta & \beta & 0 & 0 & 0 & -2\beta & 0 & 0 \\
0 & 0 & 0 & 0 & 0 & 2\beta & 2\beta & 2\beta & 0 & 0 & -\beta & 0 \\
0 & 0 & 0 & 0 & 0 & 0 & 0 & 0 & 2\beta & 2\beta & \beta & 0
\end{pmatrix} \tag{15}$$

Note that A is the transpose of the transition rate matrix and its columns sum to 0. The system in [14] can be solved numerically. We used ODEINT, which is part of the SCIPY.INTEGRATE Python library. The solution allows us to determine the probability that the system is in one of the possible states at any time given an initial condition.

Transmission Rate and Basic Reproduction Number

In this section, we will outline how the transmission rate β can be linked to the basic reproduction number R_0, assuming an SIR spreading model. We used the results in this section to derive epidemic parameter values β and μ (on static networks) that result in a given value for R_0. We assume for simplicity that i) the source node has always the average number of neighbors $\langle k \rangle$, and ii) those neighbors are not connected to each other (note that this is a strong assumption and only holds approximately for very large and sparse networks). Given assumption ii), the source node transmits the disease to a given neighbour with probability $(1 - e^{-\beta t})$, where t is the time to recovery. Consequently, under the assumption i) we can write the reproduction number R_q of the source node as $R_q = \langle k \rangle \cdot (1 - e^{-\beta t})$. Taking the expectation over all possible recovery times t yields

$$
\begin{aligned}
R_0 &\approx \int_0^{+\infty} \langle k \rangle \cdot (1 - e^{-\beta t}) \cdot \mu e^{-\mu t}\, dt \\
&= \langle k \rangle \cdot \mu \int_0^{+\infty} (e^{-\mu t} - e^{-(\mu+\beta)t})\, dt \\
&= \langle k \rangle \cdot \left(1 - \frac{\mu}{(\mu + \beta)}\right) \quad .
\end{aligned} \tag{16}
$$

If we assume without loss of generality that $\mu = 1$, we can solve the above result for β to get:

$$\beta \approx \frac{R_0 \,/\, \langle k \rangle}{1 - R_0 \,/\, \langle k \rangle} \tag{17}$$

This expression now allows us to set the transmission rate β such that a certain R_0 is achieved given a network with an average degree $\langle k \rangle$. Of course, the simulation results for R_0 will deviate slightly from the expected result due to the approximate nature of [16]. Two opposing effects play a role here: First, in real networks neighbors of a given node often exhibit some clustering and are directly or indirectly connected to each other (i.e., assumption ii) is violated), which may deplete the number of susceptible neighbors before the source node had the chance to infect them (8). As a consequence, R_0 based on simulations incurs a downward correction with respect to the approximation above. Second, in our experiments on static networks we omit all simulation runs in which the source does not infect any other nodes. This leads to an upward correction of R_0 as compared to equation [16]. Furthermore, assumption i) may lead to an up- or downward correction depending on the degree distribution of the network.

Static Network Results

For the static analysis, we run the proposed procedure on well-known network models, such as a 4-connected lattice, Erdős-Rényi (ER) networks, and networks with community structure. This fosters our understanding of the problem and allows us to analyze the effects of network characteristics, such as density or modularity, on the inference problem. For simplicity, we assume for the remainder of this section that T is known.

Source Inference on Static Network Models. The first part of the analysis is concerned with inferring the source of an epidemic spread based on the fully observed set of nodes $O_{t_1} = V$. For this we conducted 1,000 experiments for different types of networks. Each experiment consists of i) a simulation of a ground truth outbreak starting from a source node that is picked uniformly at random and restricted to have a final outbreak size of at least 10, and ii) source inference at different times based on the fully observed set of nodes. We set $\mu = 1$ (without loss of generality) and β is determined such that $R_0 \approx 2$. For all experiments we use $n = 10{,}000$ simulations to compute the MCS node state probabilities. For comparison, we also show the performance of a random inference method that selects the source estimate uniformly at random from the pool of all activated nodes.

Figure S5 shows the source inference results for four different static network models: Erdős-Rényi (ER) networks with varying size and density, community-structured networks with varying modularity Q_{mod}, a 4-connected lattice of size $N = 20 \times 20$, and a scale-free network with a degree distribution that follows a power law. The source inference performance in a single experiment is measured by the shortest path length between the true source node q_0 and the maximum-a-posteriori (MAP) node $\hat{q}$. This measure — also called error distance — has been used in other works (9, 10) and is a robust measure for source inference problems, since it does not severely penalize situations where $\hat{q}$ is in the vicinity of q_0 but cannot unequivocally identify the true source. In order to make the results for the different networks comparable, we divide the shortest path lengths by the diameter of the network. The resulting performance measure over all experiments is called the normalized average shortest path length (NASPL) and can be interpreted as the average distance between the true source and the source estimate measured in terms of the diameter of the network. Note that in some cases, especially for later inference times, NASPL is increasing to levels that are not useful from a practical perspective. This can be seen by observing that the number of nodes within an error distance of l grows roughly as $\sum_{i=1}^{l} \langle k \rangle^l$ under the assumption that the network is locally close to a regular tree. For example, a network with average degree $\langle k \rangle = 10$ will have around $\mathcal{O}(100)$ nodes that are within an error distance of $l = 2$ of the source estimate.

Panels (a–b) in Figure S5 show the results for ER networks of different sizes N, where the average degree is fixed at $\langle k \rangle = 5$. Unsurprisingly, NASPL increases with time for all three networks. Loosely speaking, the information about the source diffuses over time and inference becomes harder. As we can see in (a), the size of the network does not seem to impact the *relative* inference performance. For all three networks, mean-field-like inference is considerably better than random inference.

In Panels (c–d) we consider ER networks of fixed size $N = 500$, but vary the edge probability p in order to get networks with different densities and average degrees $\langle k \rangle = Np$. As shown in (c), a higher average degree (and hence a higher density) leads to a deterioration of the relative inference performance, despite the fact that the epidemics evolve more slowly on denser networks (see Panel (d)). Note that the slower evolution on denser networks is due to higher clustering values, which our adjustment of the transmission rate (outlined above) cannot properly take into account. Here too, the random inference is substantially worse, but the advantage of mean-field-like inference over the random method seems to be more pronounced for sparser networks.

Panels (e–f) show the results for community-structured networks. These networks were created according to the algorithm outlined in Salathé and Jones (11) and have $N = 800$ nodes in total. Each network contains 20 small-world communities ($k = 4$ and $p = 0.1$) that each consist of 40 nodes and 80 edges. We then add 400 inter-community edges and, as a result, we get a network with a total of 2,000 edges, $\langle k \rangle = 5$, and modularity (community assortativity coefficient) $Q_{mod} \approx 0.79$. By first rewiring 100 and then 200 of the inter-community edges (such that they become intra-community edges) we can increase the modularity to $Q_{mod} \approx 0.84$ and $Q_{mod} \approx 0.89$, respectively. Interestingly, a higher modularity leads to better source inference results, on average. However, it is likely that the improvement is not uniquely due to the increased modularity but also due to the epidemic evolving more slowly on graphs with higher modularity (see Panel (f)).

Panels (g) and (h) present the results for the lattice and the scale-free network, respectively. The scale-free network consists of one connected component with $N = 443$ nodes and its degree distribution follows a Zipf distribution with $\gamma = 2.1$, thus exhibiting scale-free properties. While the topology of the lattice leads to a slowly evolving epidemic, which is why we run the inference up to time $t_1 = 20$, the epidemic develops very rapidly on the scale-free network and already saturates after two time steps. The results for both networks are qualitatively similar to the results presented above. However, we find the unexpected

effect that the inference performance for the scale-free network does not decrease monotonically with time but in fact improves slightly for later times.

Active Querying on Static Network Models. The second part of the analysis is concerned with the active querying problem. The experimental setup is similar to the previous section. After generating a ground truth outbreak with the same specifications as above, we conduct the inference and querying process at a given time t_1. The process starts with picking one node uniformly at random, as the first observed node, from all activated nodes. Then, the inference and querying are performed iteratively until no more nodes are left to be queried or up to a specified number of queries. At each inference step, we measure the performance by means of NASPL.

First, we are interested in comparing the performance of the three active querying strategies. The results of this analysis are presented in Figs. S6, S7, S8, S9, S10. It is apparent from these Figures that AKLD rarely performs worse than the other two active querying strategies (the one notable exception is the scale-free network during the early phase of an outbreak). More importantly, AKLD seems to outperform the other strategies in many cases, especially at intermediate inference times. Therefore, we will focus on the AKLD strategy in the remainder of this section.

We now want to compare AKLD to the two baseline strategies ONE-HOP and RANDOM. Figure S11 presents the results of this analysis. Since the networks are of different sizes, we query 10% of all nodes in each network. What stands out is that AKLD's performance is at least comparable to and often better than that of ONE-HOP and/or RANDOM. AKLD generally reaches the full inference performance within 10% of queried nodes, with one exception being the lattice for later times. For the scale-free network, the performance of AKLD is even slightly better than the full inference performance for later times. One possible explanation for this phenomenon was found by Paluch et al. (12) who state that the information we receive from nodes that connect to the true source via network hubs can be misleading and, as a consequence, we may observe a degradation of the source inference performance in scale-free networks. Our AKLD strategy seems to be a way of avoiding this trap by focusing on a small fraction of informative nodes.

We also tested how varying the network characteristics, such as size, density, and modularity, influences the querying (see Figure S12). Overall, we did not find a substantial effect of those network characteristics on the (AKLD) querying results, and differences in the learning curves seem to rather reflect varying levels in the overall difficulty to infer the source. Note, however, that querying only 10 nodes in the ER network of size $N = 100$ (i.e., 10%) leads to substantially worse results as compared to larger networks. A priori, we would have expected the relative querying performance to be similar for the three networks of different sizes.

In summary, these results show that AKLD is in most cases the best querying strategy. However, the differences between AKLD and the other strategies are less pronounced than for the empirical networks we consider in the main paper. The real strength of AKLD only appears on sparser networks which we cannot generate with the static network models (and network sizes) we used.

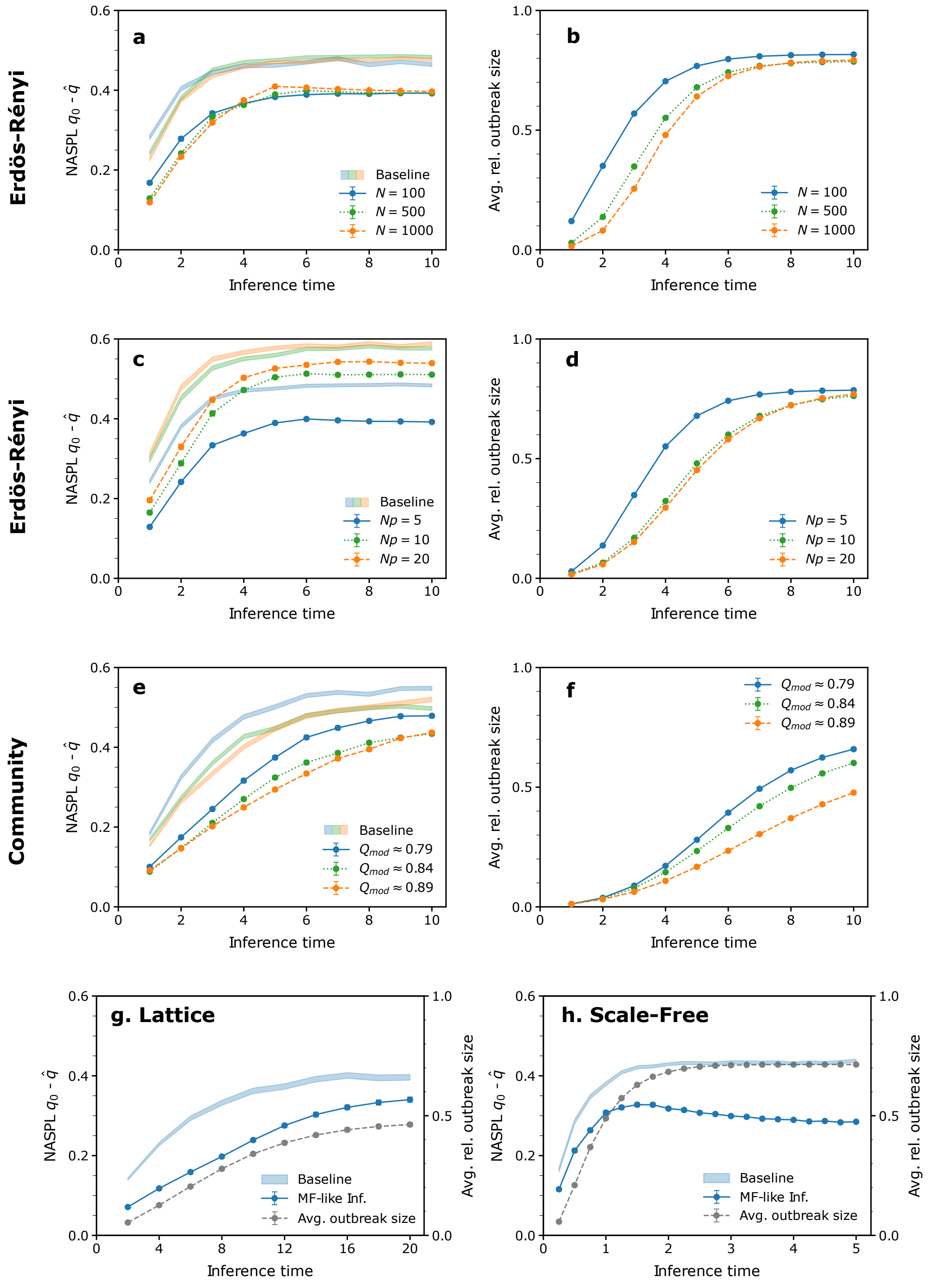

Fig. S5. The source (mean-field-like) inference results over time if the states of all nodes are observed. (a) Normalized average shortest path length (NASPL) between true source q_0 and $\hat{q}$ for ER networks of different sizes $N = \{100,\ 500,\ 1000\}$. The shaded areas show the performance of the random inference method (baseline). (b) Average relative outbreak sizes for the three different networks in Panel (a). (c–d) Similar to (a–b), but they show the results for ER networks with different average degree $Np = \{5,\ 10,\ 20\}$, keeping N fixed at 500. (e–f) Results for community-structured networks of varying modularity $Q_{mod} = \{0.79,\ 0.84,\ 0.89\}$. (g) Inference performance for 4-connected lattice ($N = 20 \times 20$) on left axis. Corresponding average relative outbreak size on right axis. (h) Similar to (g) but for scale-free network ($N = 443$) with power $\gamma = 2.1$. In all Panels, error areas and error bars represent $\pm$ one standard error (s.e.m.), but errors are small and often hardly discernible.

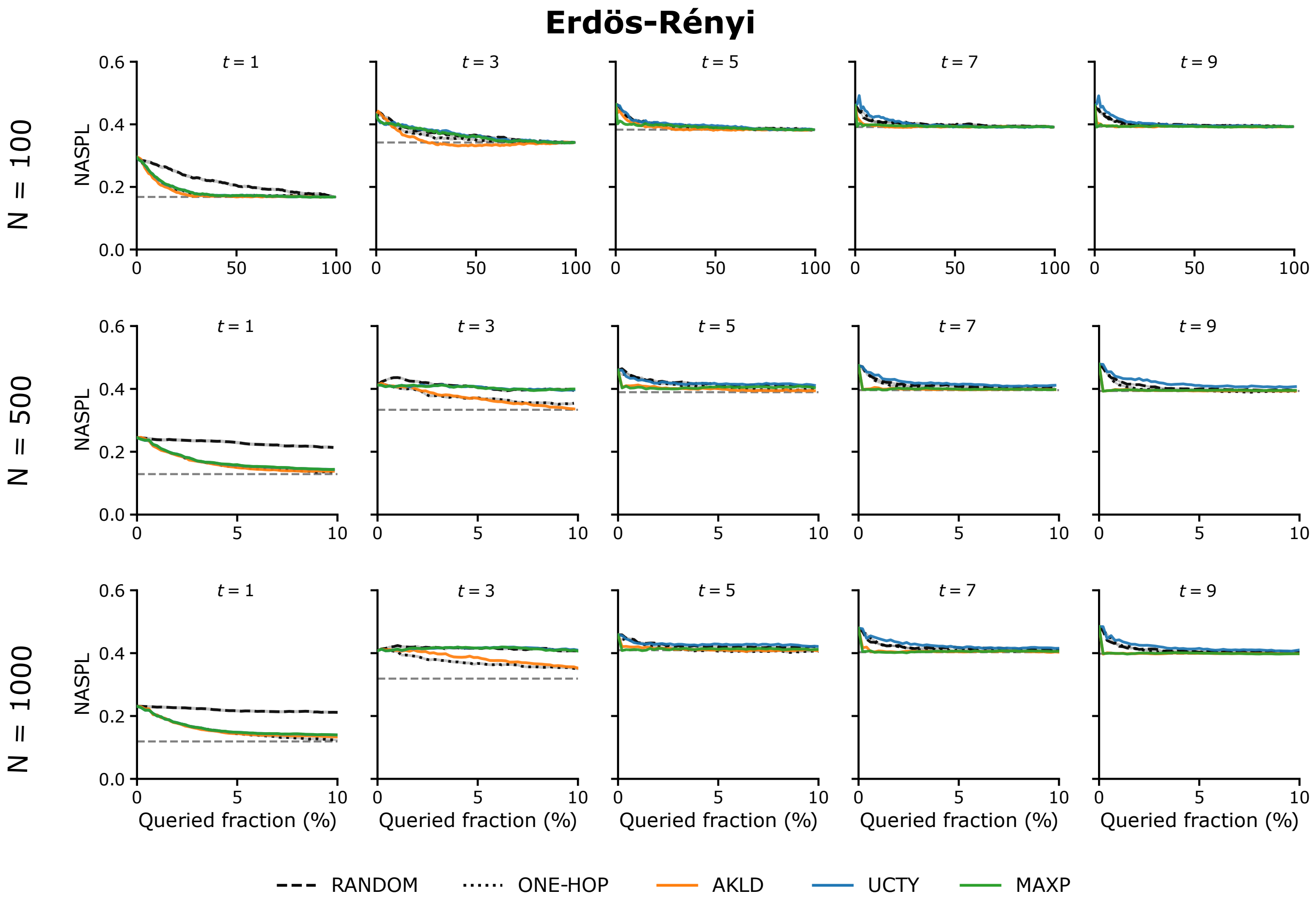

Fig. S6. Active querying (all strategies) as compared to baseline querying strategies on Erdős-Rényi (ER) networks of different size. The first, second, and third row correspond to network sizes of $N = 100, 500, 1000$, respectively. All networks have average degree $\langle k \rangle = 5$. For each network, we show the querying results at different times $t_1 = 1, 3, 5, 7, 9$. The performance of the different strategies is measured by the normalized average shortest path length (NASPL) between the true source q_0 and the source estimate $\hat{q}$. For each setting, we ran a total of $1{,}000$ experiments and we used the spreading parameters $\beta = 0.67$ and $\mu = 1$ such that $R_0 \approx 2$. Error areas represent $\pm$ one standard error (s.e.m.). The dashed horizontal line represents the inference performance when all nodes are observed.

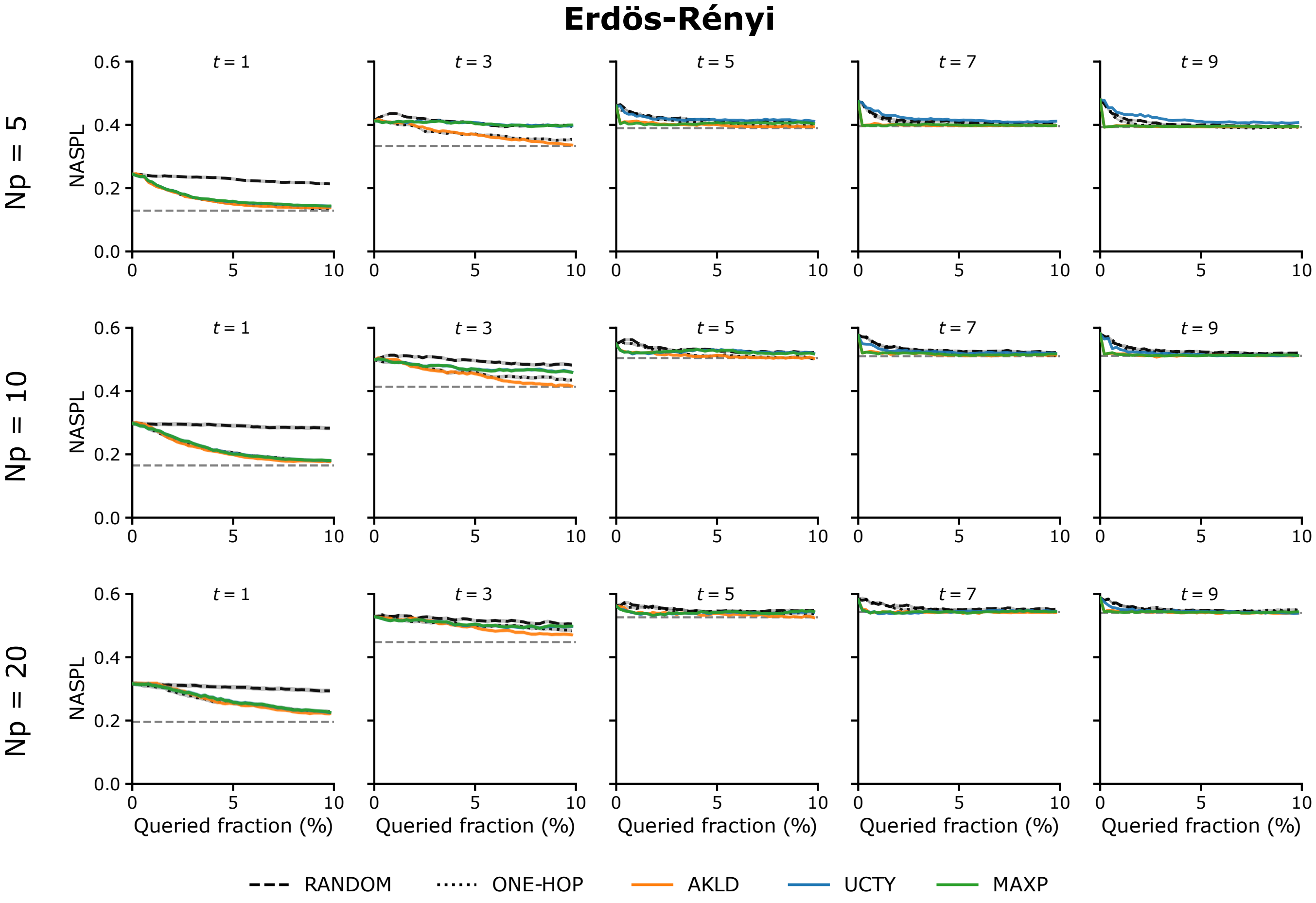

Fig. S7. Active querying (all strategies) as compared to baseline querying strategies on Erdős-Rényi (ER) networks of different average degree (density). The first, second, and third row correspond to average degrees of $Np = 5, 10, 20$, respectively. All networks have a size $N = 500$. For each network, we show the querying results at different times $t_1 = 1, 3, 5, 7, 9$. The performance of the different strategies is measured by the normalized average shortest path length (NASPL) between the true source q_0 and the source estimate $\hat{q}$. For each setting, we ran a total of $1{,}000$ experiments. The recovery rate μ was set to 1 and β was adjusted depending on the average degree such that $R_0 \approx 2$. Error areas represent $\pm$ one standard error (s.e.m.). The dashed horizontal line represents the inference performance when all nodes are observed.

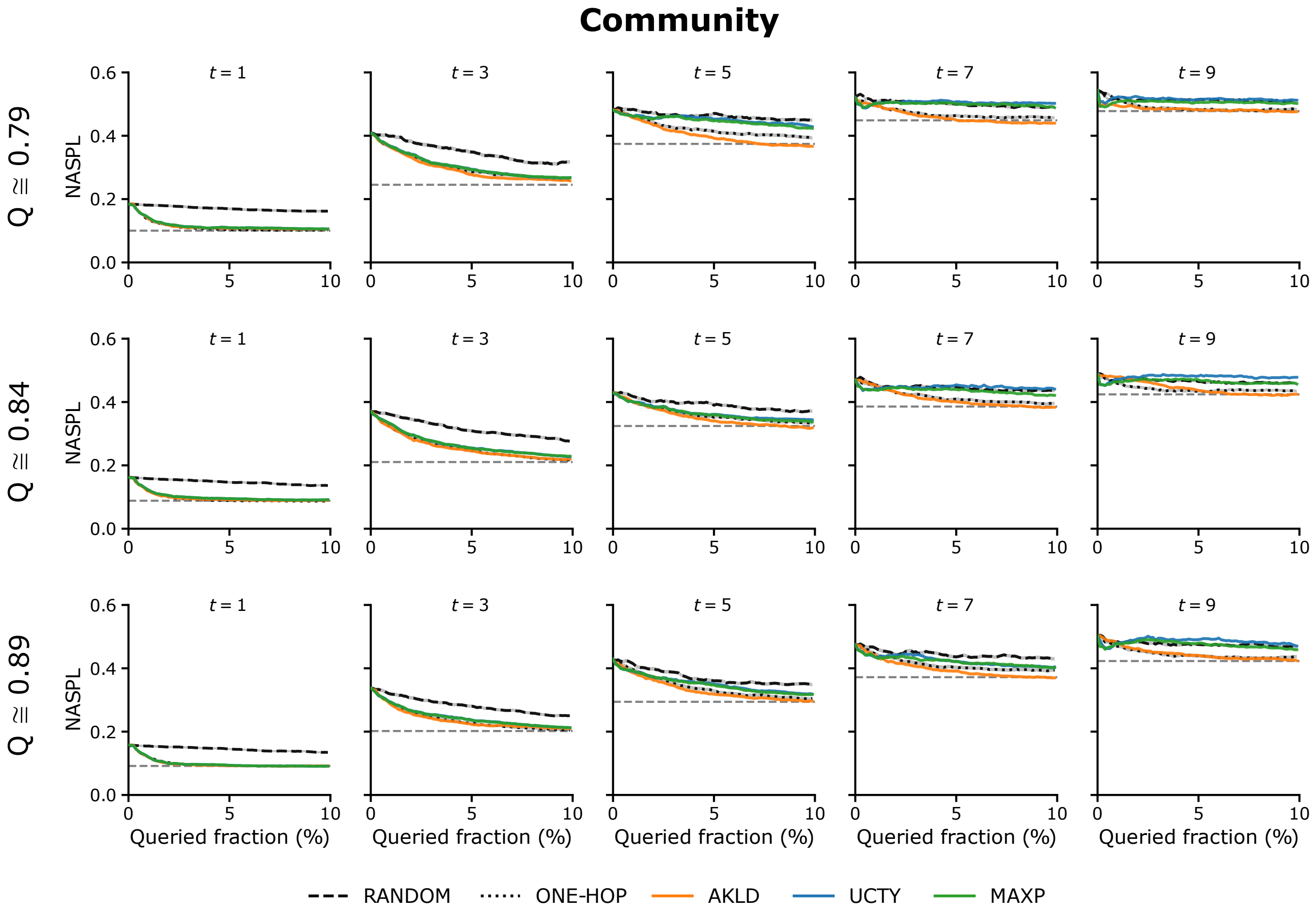

Fig. S8. Active querying (all strategies) as compared to baseline querying strategies on community networks with varying modularity. The first, second, and third row correspond to modularity values of $Q_{mod} = 0.79, 0.84, 0.89$, respectively. All networks have average degree $\langle k \rangle = 5$ and are of size $N = 800$. For each network, we show the querying results at different times $t_1 = 1, 3, 5, 7, 9$. The performance of the different strategies is measured by the normalized average shortest path length (NASPL) between the true source q_0 and the source estimate $\hat{q}$. For each setting, we ran a total of $1{,}000$ experiments and we used the spreading parameters $\beta = 0.67$ and $\mu = 1$ such that $R_0 \approx 2$. Error areas represent $\pm$ one standard error (s.e.m.). The dashed horizontal line represents the inference performance when all nodes are observed.

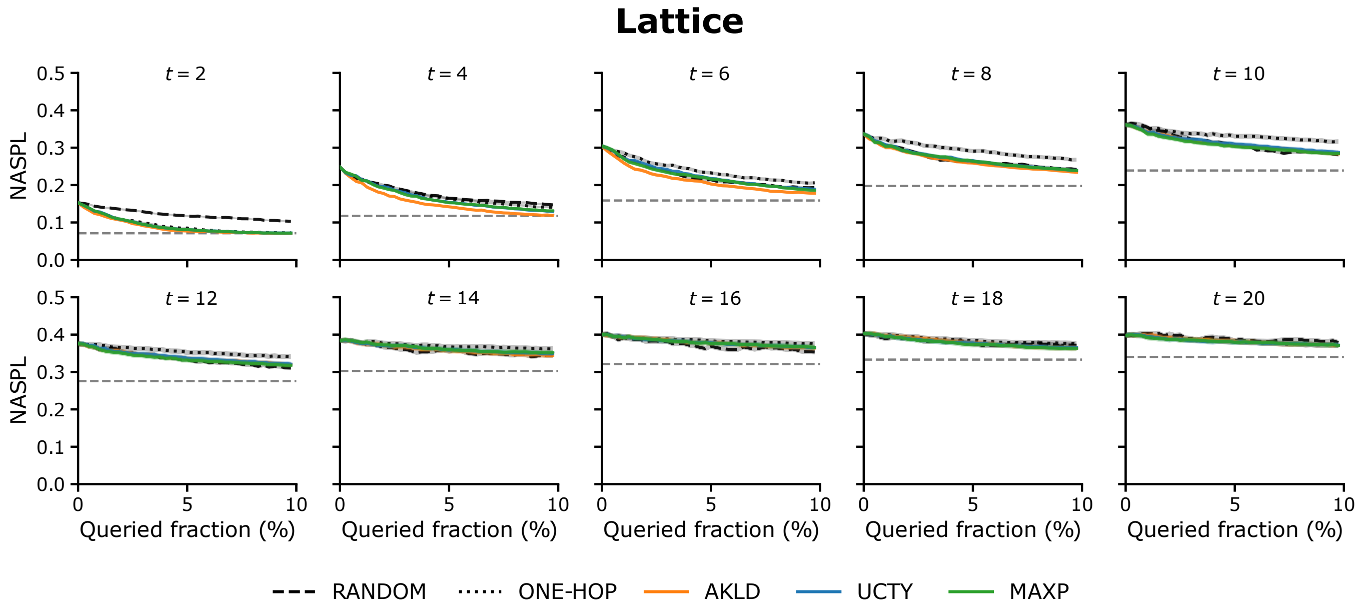

Fig. S9. Active querying (all strategies) as compared to baseline querying strategies on 4-connected lattice network of size $N = 20 \times 20$. We show the querying results at different times $t_1 = 2, \ldots, 20$. The performance of the different strategies is measured by the normalized average shortest path length (NASPL) between the true source q_0 and the source estimate $\hat{q}$. The results are based on a total of $1{,}000$ experiments and we used the spreading parameters $\beta = 1$ and $\mu = 1$ such that $R_0 \approx 2$. Error areas represent $\pm$ one standard error (s.e.m.). The dashed horizontal line represents the inference performance when all nodes are observed.

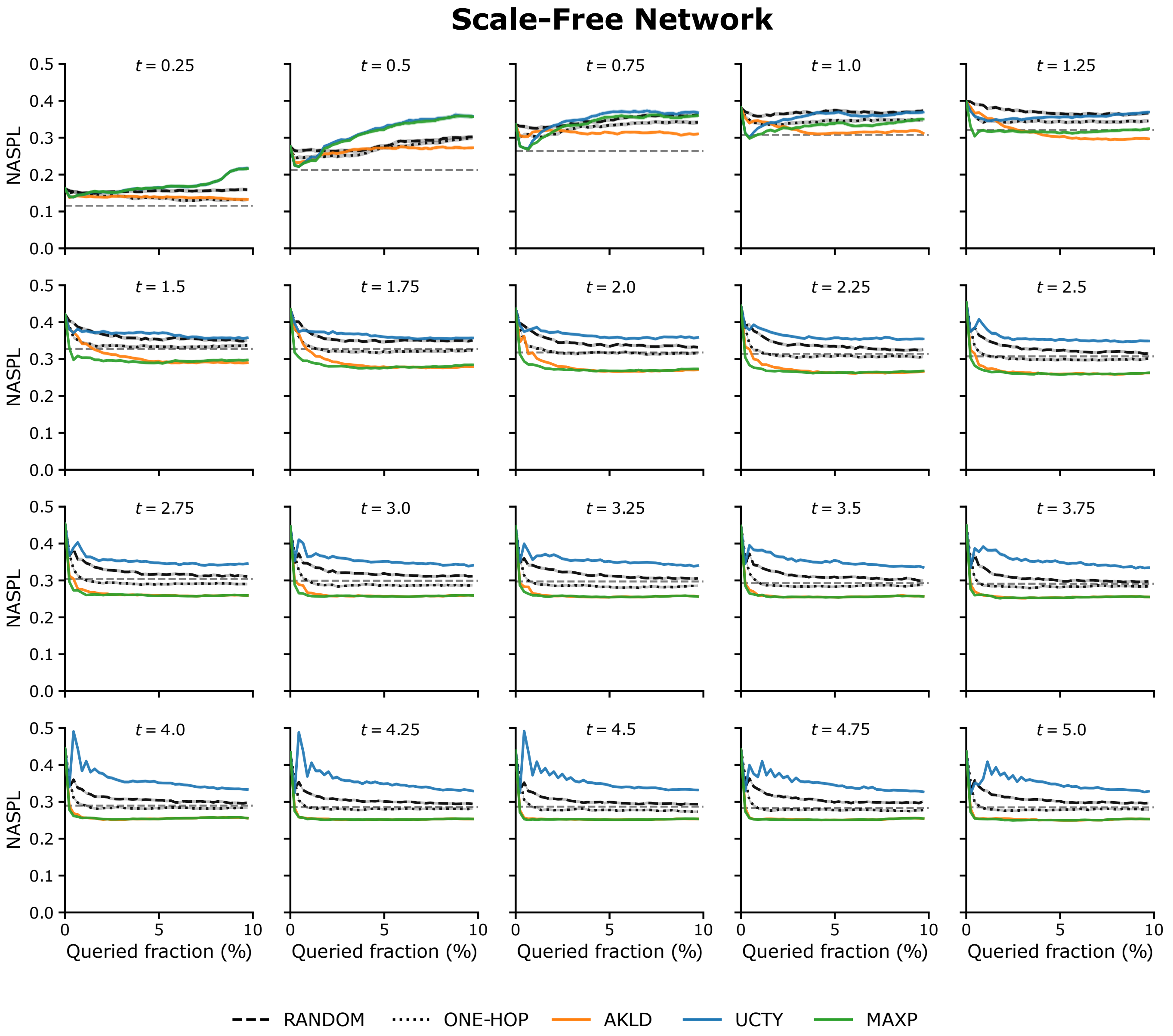

Fig. S10. Active querying (all strategies) as compared to baseline querying strategies on scale-free network of size $N = 443$ with the degree distribution following a Zipf distribution with $\gamma = 2.1$. We show the querying results at different times $t_1 = 0.25, \ldots, 5$. The performance of the different strategies is measured by the normalized average shortest path length (NASPL) between the true source q_0 and the source estimate $\hat{q}$. The results are based on a total of $1{,}000$ experiments and we used the spreading parameters $\beta = 1.76$ and $\mu = 1$ such that $R_0 \approx 2$. Error areas represent $\pm$ one standard error. The dashed horizontal line represents the inference performance when all nodes are observed.

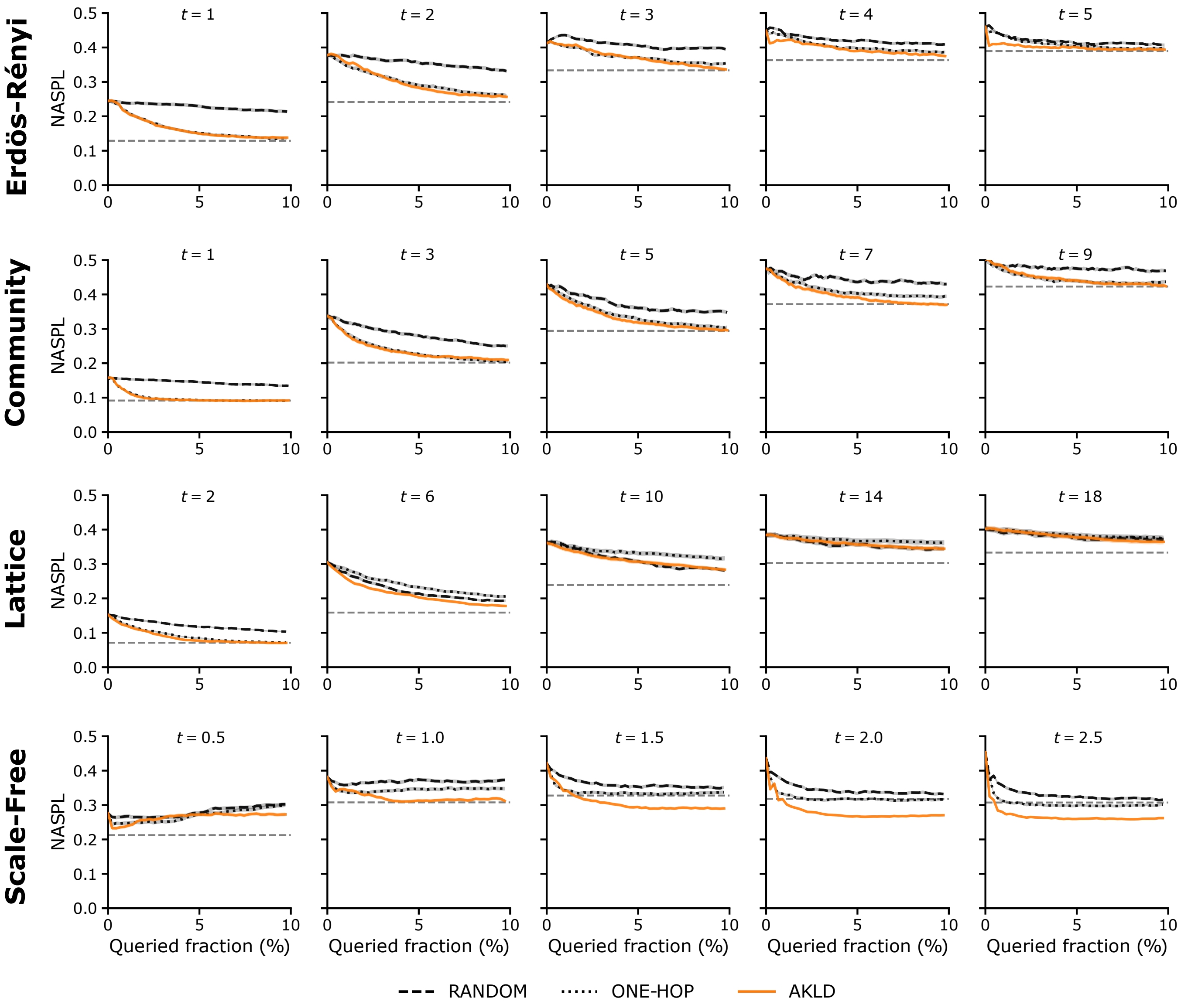

Fig. S11. The AKLD strategy as compared to the baseline querying strategies. The first row shows the results for the ER network of size $N = 500$ and average degree $Np = 5$. The second row shows the results for a community-structured network of size $N = 800$ and modularity $Q_{mod} = 0.89$. The third row shows the results for the 4-connected lattice of size $N = 20 \times 20$ and the fourth row shows the results for a scale-free network of size $N = 443$ with the degrees following a Zipf distribution with $\gamma = 2.1$. The horizontal dashed lines indicate the full inference performance. All curves contain error areas that represent $\pm$ one standard error (s.e.m.). Note that the times shown in the plot were selected depending on the speed with which the epidemics evolve on the different networks.

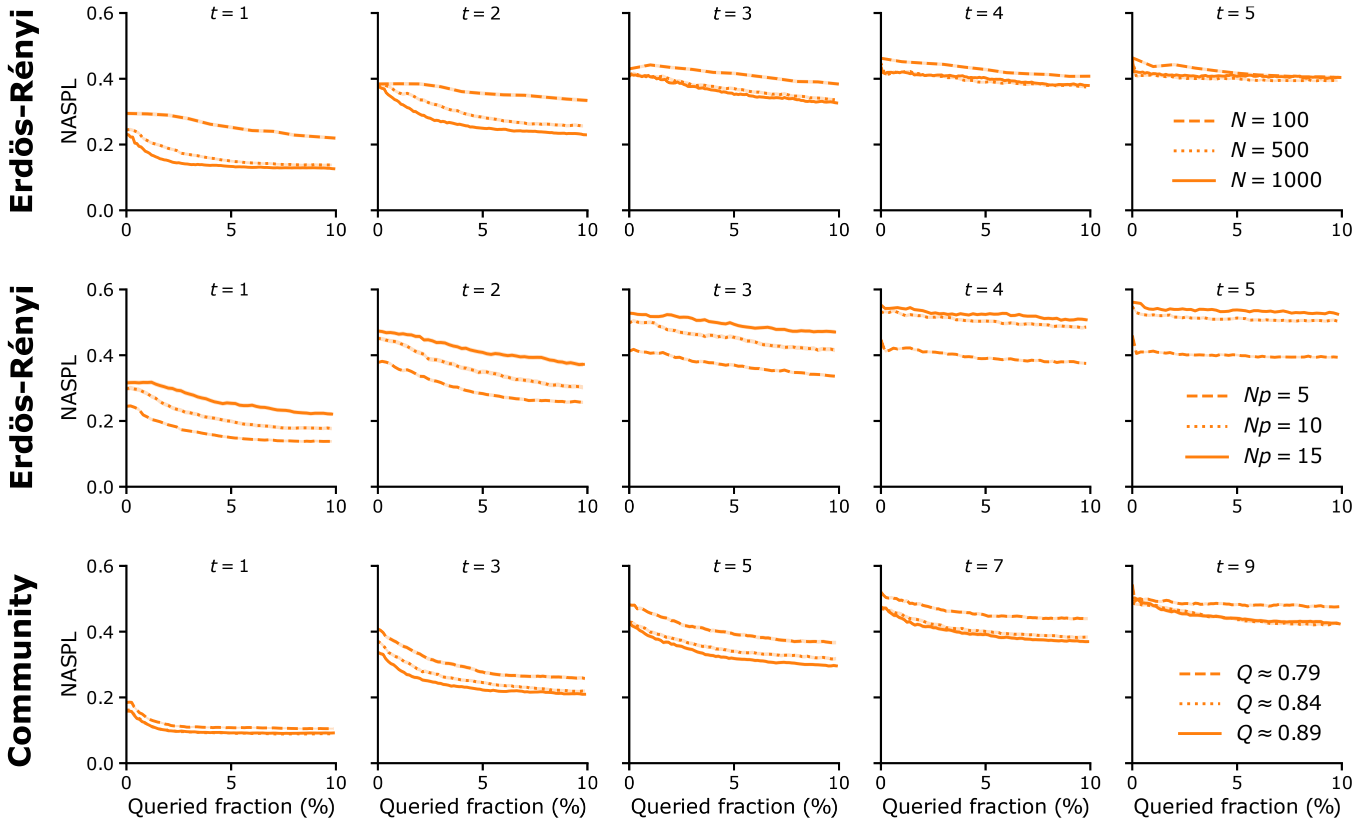

Fig. S12. AKLD strategy results for Erdős-Rényi (ER) networks of different size and different average degree (density), and for community networks of different modularity. Each curve shows the AKLD querying performance for a certain value of the network property under consideration. For the ER networks, we show the results at times $t_1 = 1, \ldots, 5$. For the community networks, we present the results at times $t_1 = 1, \ldots, 9$ because the epidemics evolves less fast than on ER networks. The performance of the different strategies is measured by the normalized average shortest path length (NASPL) between the true source q_0 and the source estimate $\hat{q}$. Error areas represent $\pm$ one standard error (s.e.m.).

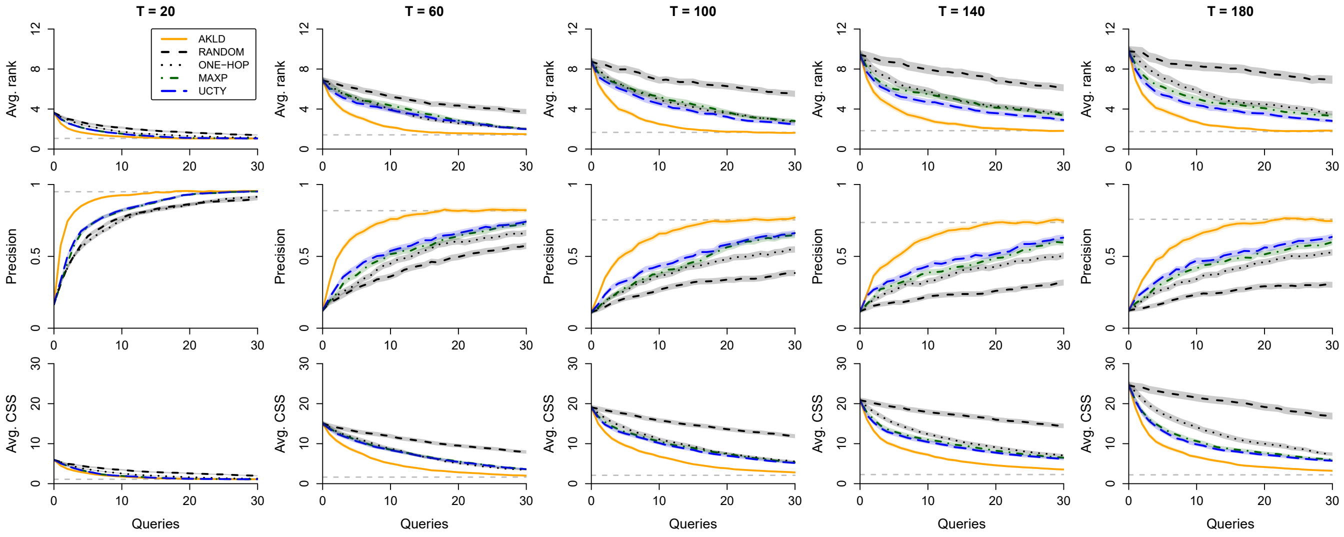

Fig. S13. Performance of all querying strategies for the **PIG** network and $R_0 = 1.5$. The first, second, and third row of plots show the average rank, precision, and average CSS, respectively. The columns show the results for different T. The horizontal dashed lines indicate the full inference performance. All curves contain error areas that represent $\pm$ one standard error (s.e.m.).

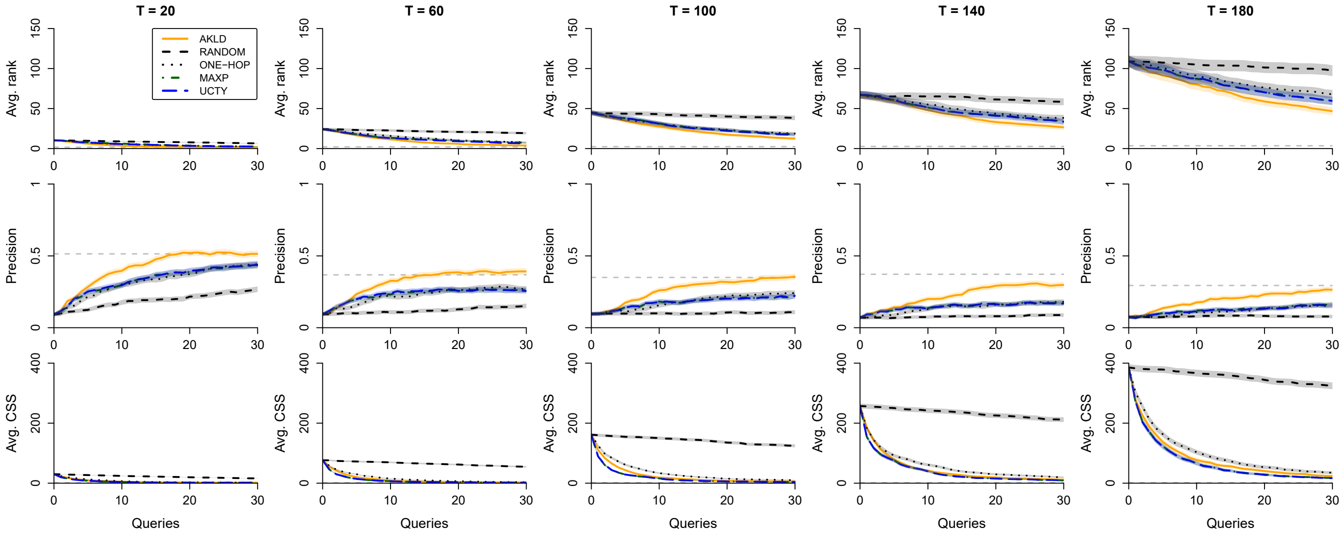

Fig. S14. Performance of all querying strategies for the **ESCORT** network and epidemic parameters $\beta = 0.3$ and $\mu = 0.01$. The first, second, and third row of plots show the average rank, precision, and average CSS, respectively. The columns show the results for different T. The horizontal dashed lines indicate the full inference performance. All curves contain error areas that represent $\pm$ one standard error (s.e.m.).

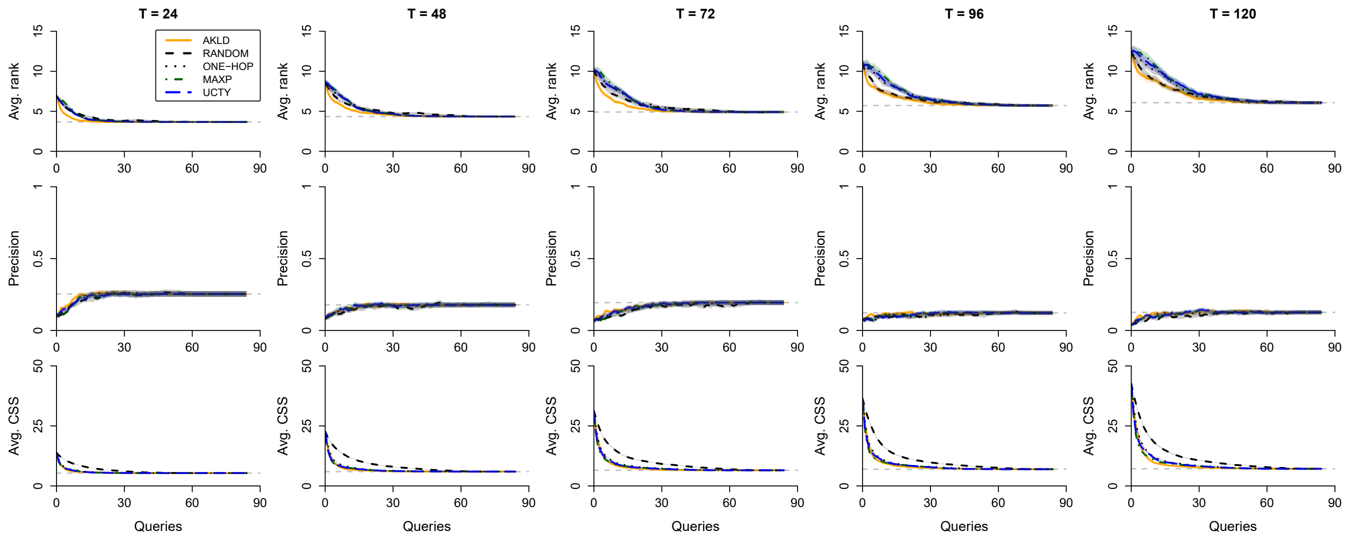

Fig. S15. Performance of all querying strategies for the **MALAWI** network and $R_0 = 1.5$. The first, second, and third row of plots show the average rank, precision, and average CSS, respectively. The columns show the results for different T. The horizontal dashed lines indicate the full inference performance. All curves contain error areas that represent $\pm$ one standard error (s.e.m.).

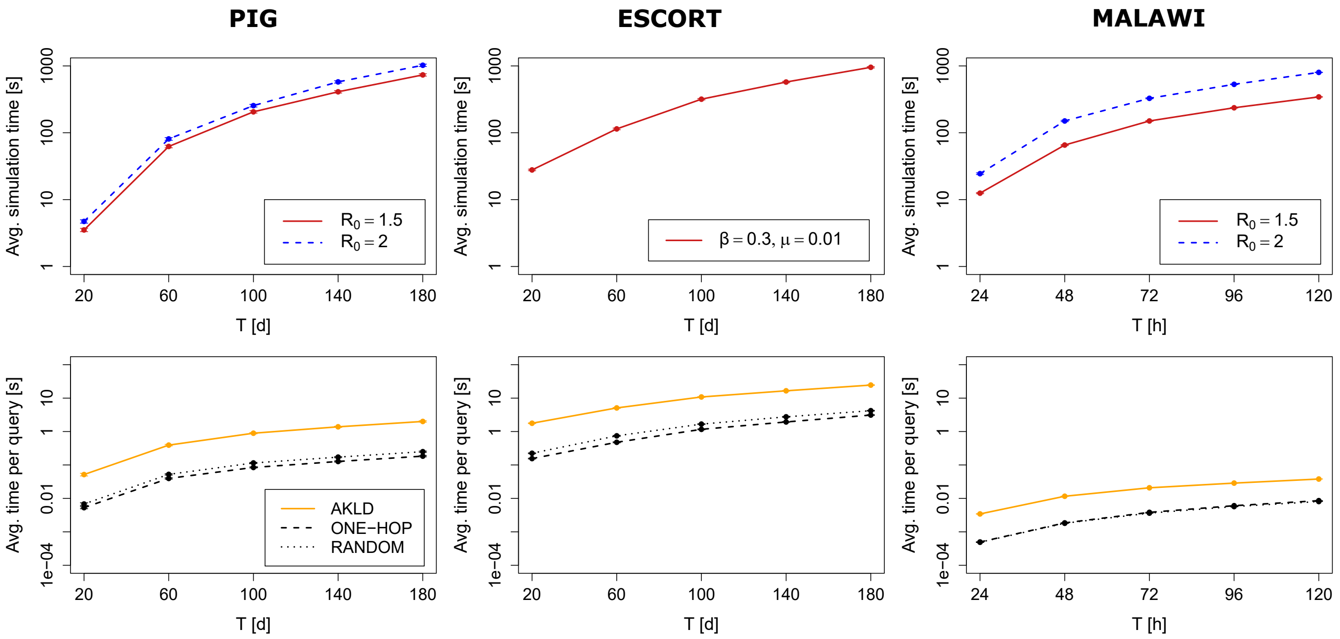

Fig. S16. CPU run times for the simulation procedure and the querying on all three networks. The Panels in the top row show the average CPU time (in seconds) consumed by the simulation procedure for different durations T. For the PIG and the MALAWI, we show curves for $R_0 = 1.5$ and $R_0 = 2$. Note that the run times not only depend on the size of the network N or the number of source-duration pairs but in particular also on how sparse the contact structure is. This is why the average run times for the MALAWI network are comparatively high, even though it is a small network with only $N = 86$ nodes. The Panels in the bottom row show the average CPU time (in seconds) consumed by one query using the AKLD, the ONE-HOP, or the RANDOM querying strategy. Only results for $R_0 = 1.5$ are shown and the average query times of MAXP and UCTY are similar to the baseline strategies and are thus omitted. Error bars represent $\pm$ one standard error (s.e.m.) but are small and thus hardly discernible.

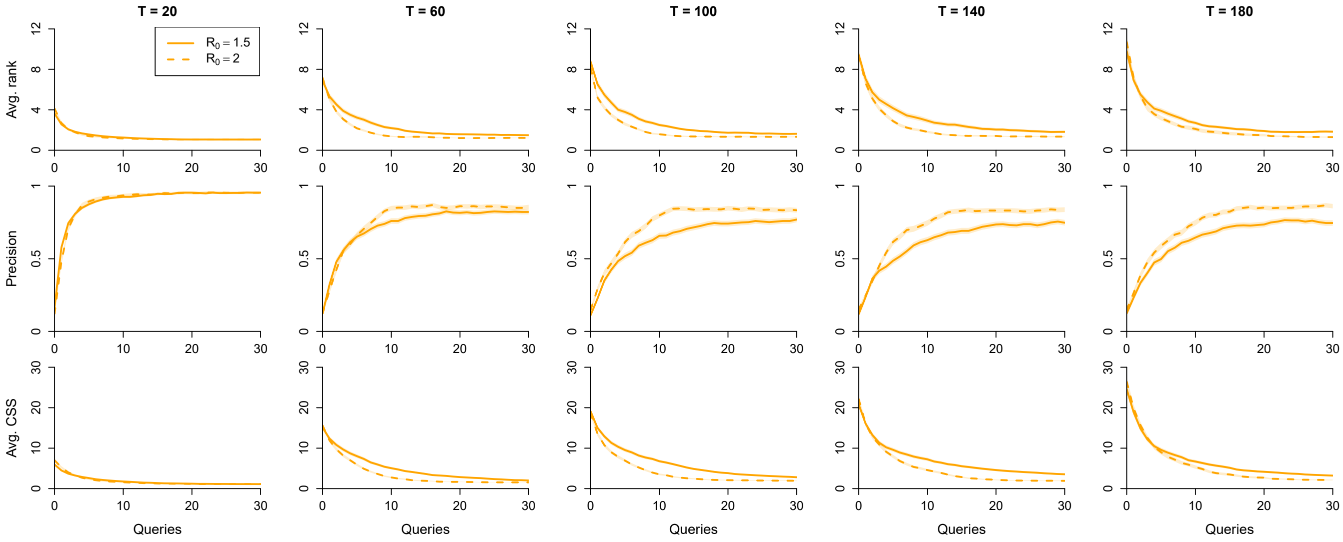

Fig. S17. The performance of the AKLD strategy on the **PIG** network and for $R_0 = 1.5$ and $R_0 = 2$. The first, second, and third row of plots show the average rank, precision, and average CSS, respectively. The columns show the results for different T. All curves contain error areas that represent $\pm$ one standard error (s.e.m.).

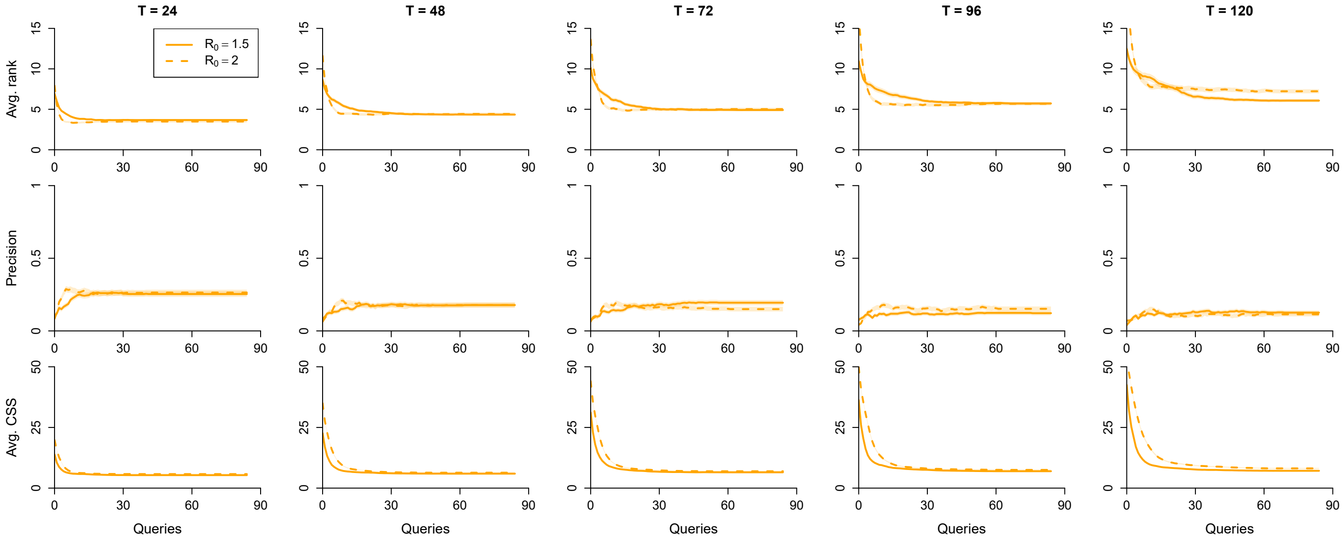

Fig. S18. The performance of the AKLD strategy on the **MALAWI** network and for $R_0 = 1.5$ and $R_0 = 2$. The first, second, and third row of plots show the average rank, precision, and average CSS, respectively. The columns show the results for different T. All curves contain error areas that represent $\pm$ one standard error (s.e.m.).

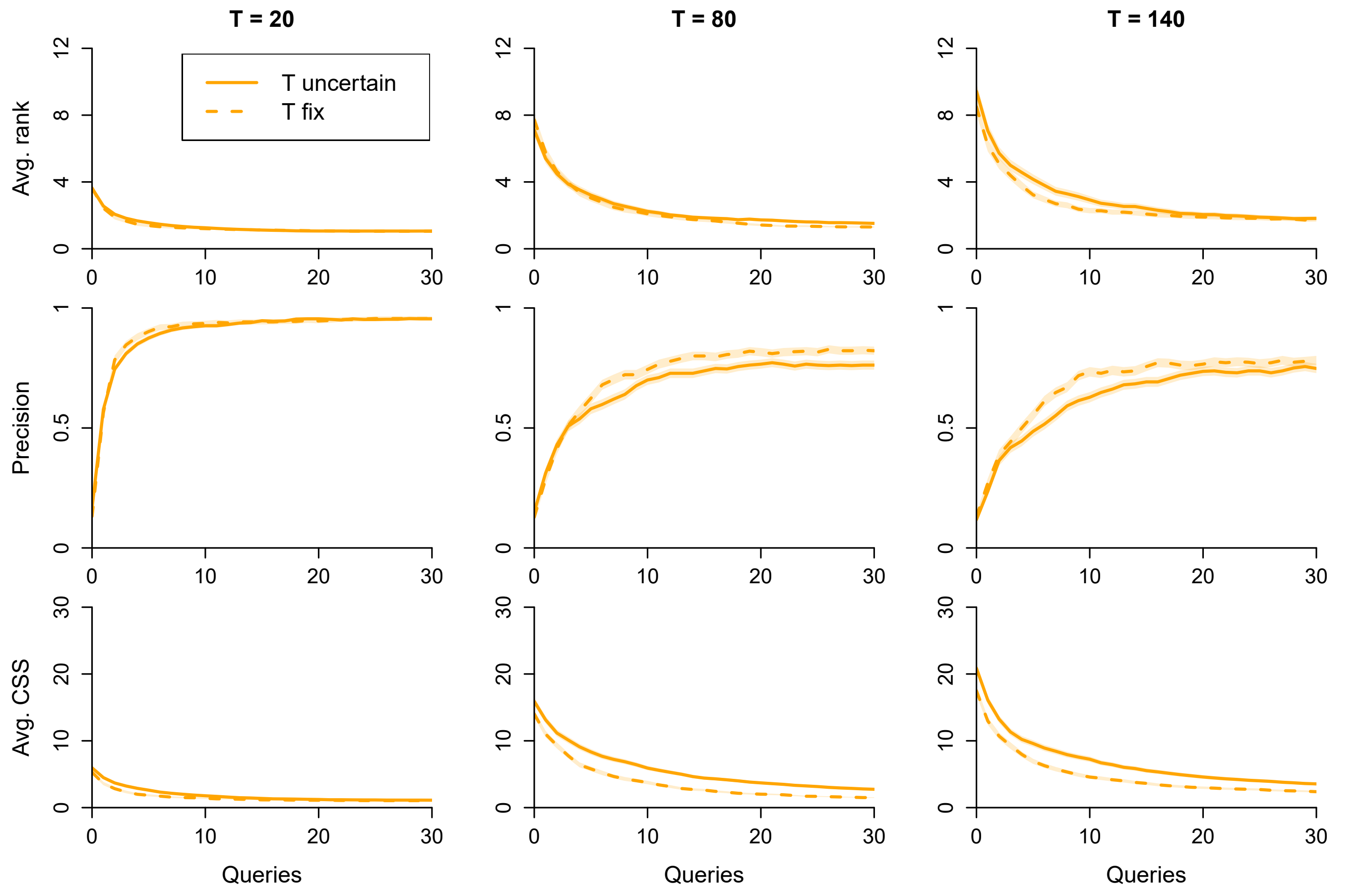

Fig. S19. The performance of the AKLD strategy on the **PIG** network when T is assumed to be known as compared to T being uncertain. The first, second, and third row of plots show the average rank, precision, and average CSS, respectively. The columns show the results for three different T. All curves contain error areas that represent $\pm$ one standard error (s.e.m.).

References

1. KP Murphy, *Machine Learning: A Probabilistic Perspective.* (The MIT Press), (2012).
2. KJ Sharkey, Deterministic epidemiological models at the individual level. *J. Math. Biol.* **57**, 311–331 (2008).
3. M Youssef, C Scoglio, An individual-based approach to sir epidemics in contact networks. *J. Theor. Biol.* **283**, 136–144 (2011).
4. IZ Kiss, JC Miller, PL Simon, *Mathematics of Epidemics on Networks.* (Springer International Publishing AG, Cham, Switzerland), 1st edition, (2017).
5. LEC Rocha, N Masuda, Individual-based approach to epidemic processes on arbitrary dynamic contact networks. *Sci. Reports* **6**, 31456 (2016).
6. AY Lokhov, M Mézard, H Ohta, L Zdeborová, Inferring the origin of an epidemic with a dynamic message-passing algorithm. *Phys. Rev. E* **90**, 012801 (2014).
7. EoN Module (https://epidemicsonnetworks.readthedocs.io/en/latest/EoN.html) (n.d.) Accessed: 2022-01-05.
8. P Holme, Fast and principled simulations of the sir model on temporal networks. *PLOS ONE* **16**, 1–15 (2021).
9. N Antulov-Fantulin, A Lančić, T Šmuc, H Štefančić, M Šikić, Identification of patient zero in static and temporal networks: Robustness and limitations. *Phys. Rev. Lett.* **114**, 248701 (2015).
10. J Jiang, S Wen, S Yu, Y Xiang, W Zhou, Identifying propagation sources in networks: State-of-the-art and comparative studies. *IEEE Commun. Surv. Tutorials* **19**, 465–481 (2017).
11. M Salathé, JH Jones, Dynamics and control of diseases in networks with community structure. *PLOS Comput. Biol.* **6**, 1–11 (2010).
12. R Paluch, X Lu, K Suchecki, BK Szymański, JA Hołyst, Fast and accurate detection of spread source in large complex networks. *Sci. Reports* **8**, 2508 (2018).
